# ﻿Comparative morphology and key to Amydetinae genera, with description of three new firefly species (Coleoptera, Lampyridae)

**DOI:** 10.3897/zookeys.1114.77692

**Published:** 2022-07-27

**Authors:** Lucas Campello, Stephanie Vaz, José R. M. Mermudes, André L. D. Ferreira, Luiz F. L. Silveira

**Affiliations:** 1 Programa de Pós-graduação em Biodiversidade e Biologia Evolutiva–Laboratório de Entomologia, Departamento de Zoologia, Instituto de Biologia, Universidade Federal do Rio de Janeiro, A1–107, Bloco A, Av. Carlos Chagas Filho, 373, Cidade Universitária, Ilha do Fundão, Rio de Janeiro, RJ, Brazil; 2 Laboratório de Entomologia, Departamento de Zoologia, Instituto de Biologia, Universidade Federal do Rio de Janeiro, A1-107, Bloco A, Av. Carlos Chagas Filho, 373, Cidade Universitária, Ilha do Fundão, Rio de Janeiro, RJ, Brazil; 3 Programa de Pós-graduação em Ecologia, Laboratório de Ecologia de Insetos, Departamento de Ecologia, Instituto de Biologia, Universidade Federal do Rio de Janeiro, A0-111, Bloco A, Av. Carlos Chagas Filho, 373, Cidade Universitária, Ilha do Fundão, Rio de Janeiro, RJ, Brazil; 4 Programa de Pós-graduação em Ecologia, Laboratório de Polychaeta, Departamento de Zoologia, Instituto de Biologia, Universidade Federal do Rio de Janeiro, A0-108, Bloco A, Av. Carlos Chagas Filho, 373, Cidade Universitária, Ilha do Fundão, Rio de Janeiro, RJ, Brazil; 5 Department of Biology, Western Carolina University, Apodaca Science Building, 1 University Drive, Cullowhee, NC 28723, USA

**Keywords:** *Amydetes*, Atlantic Forest, *Magnoculus*, *Memoan*, Neotropics

## Abstract

Amydetinae is an exclusively Neotropical subfamily of fireflies, distributed among three genera: *Amydetes* Illiger, 1807, *Magnoculus* McDermott, 1964, and *Memoan* Silveira & Mermudes, 2013. Here, we describe three new species of Amydetinae: two belonging to *Amydetes* (*A.alexi***sp. nov.** and *A.marolae***sp. nov.**) and one to the previously monotypic *Memoan* (*Me.conani***sp. nov.**). All three species are known only from the Atlantic Forest in southeastern Brazil. Endoskeletal structures of *Memoan* and *Magnoculus* species are described and compared with those of *Amydetes* for the first time. After studying the type material, *Photinusfruhstorferi* Pic, 1942 is transferred to *Memoan*, generating *Memoanfruhstorferi***comb. nov.**, and *Me.ciceroi* Silveira & Mermudes, 2013 **syn. nov.** is placed as a junior synonym. We also redescribe *Magnoculusobscurus* Olivier, 1885 and compare it to other species of genus and to other amydetine taxa to identify potential new diagnostic traits for the Amydetinae and its constituting genera. We provide an updated diagnosis for *Memoan*, illustrations for all four species, and a distribution map for the three new species, as well as a key to adult males of the three amydetine genera, and an updated key to *Amydetes* species based on males.

## ﻿Introduction

Amydetinae (sensu Martin et al. 2019) is an exclusively Neotropical firefly subfamily that includes nearly 50 known species in three genera: *Amydetes* Illiger, 1807; *Magnoculus* McDermott, 1964; and *Memoan* Silveira & Mermudes, 2013. The Amydetinae are only known from male specimens, and females remain unknown despite extensive sampling with passive collection methods (e.g., Silveira et al. 2020), which suggests they might be flightless and possibly larviform (see McDermott 1964; Silveira and Mermudes 2013, 2014a). Male Amydetinae are diagnosed by the following combination of traits: continuous glow; pleural ventral suture visible; eyes ventrally close-set; punctures wide (distance among punctures smaller than puncture diameter) and with irregular outlines on pronotum and scutellum; and absence of tibial spurs (Silveira and Mermudes 2013; Martin et al. 2019).

Martin et al. (2019) were the first to perform a phylogenetic analysis to recover *Amydetes* as sister to *Memoan*, a placement previously suggested by Silveira and Mermudes (2013) based on morphological similarities. However, Martin et al. (2019) did not include *Magnoculus*, and the monophyly of Amydetinae sensu Martin et al. (2019) has yet to be tested, as the three genera were never included in the same phylogenetic analysis. *Magnoculus* has been associated with the genus *Cheguevaria* Kazantsev, 2006 (Jeng 2008; Martin et al. 2017), whose placement within Lampyridae remains elusive (Ferreira et al. 2019).

*Amydetes* has 21 species across South America (records from Mexico have been disputed: Silveira and Mermudes 2014a; Pérez-Hernández et al. 2022). Of these, 13 were described in a recent taxonomic review, mostly from museum specimens, suggesting that the diversity is underestimated due to lack of studies and comprehensive, extensive, and targeted sampling (Silveira and Mermudes 2014a). *Magnoculus* has 28 known species, distributed from southern Mexico to Argentina (McDermott 1966; Constantin 2011; Pérez-Hernández et al. 2022). The relatively recent discovery of many newly described species indicates that species diversity is also perhaps underestimated (Constantin 2011). However, the lack of revisions and redescriptions makes it difficult to identify *Magnoculus* species, especially in Brazil, where this genus is most diverse.

Both *Amydetes* and *Magnoculus* males have flabellate antennae, but the former has between 23 or more antennomeres, while the latter has only 11 (Constantin 2011; Nunes et al. 2020). McDermott (1964: key on page 12) mentioned *Magnoculus* species with more than 14 antennomeres, but these were, to our knowledge, never described or illustrated. Finally, the monotypic *Memoan* is unique among the Amydetinae for its serrate antenna with 10 antennomeres. This genus was placed as Lampyridae*incertae sedis* due to the unique combination of traits seen among several subfamilies (i.e., incompletely divided last antennomere, apical maxillary palpomere digitiform, absence of tibial spurs, and continuous glow), but later placed in Amydetinae, supported by phylogenomic data (Martin et al. 2019). *Memoan* remains known only from the type locality of the type species, in the state of Espírito Santo, southeastern Brazil.

The lack of zoological studies contrasts with the biotechnological value and potential of the Amydetinae, as some species have been the source of materials for applied research (e.g., Oliveira and Viviani 2019; Pelentir et al. 2021). For instance, bioluminescent molecules from *Amydetesvivianii* Silveira & Mermudes, 2014a have been used in ratiometric biosensing of temperature and pH in cells (Oliveira and Viviani 2019). Therefore, taxonomic tools and resources to facilitate amydetine identification, as well as more intensive sampling and field observations are sorely needed to support applied research.

The amydetines are particularly rich in the Brazilian Atlantic Forest, the second largest tropical forest on the American continent and one of the 25 global biodiversity hotspots (Norman 2003). Several lampyrid genera are considered endemic to the Atlantic Forest, including *Memoan*, *Ybytyramoan* Silveira & Mermudes, 2014b; *Luciuranus*Silveira et al., 2016a; *Scissicauda*Silveira et al., 2016b; *Araucariocladus* Silveira & Mermudes, 2017; *Uanauna*Campello-Gonçalves et al., 2019; and *Costalampys*Silveira et al., 2021; which underlines the importance of this biome to understand the diversity and evolution of fireflies.

The Brazilian Atlantic Forest is a global biodiversity hotspot (Myers et al. 2000) and is particularly rich in firefly species (Silveira et al. 2020). This biome extends over more than 27° of latitude, with wide variations in relief, which contributes to the maintenance of its rich biodiversity (Oliveira-Filho and Fontes 2000). Here, we describe three new species of Amydetinae, apparently endemic to the Brazilian Atlantic Forest, and report biological observations for each. *Amydetesmarolae* sp. nov. is described from the continental island of Ilha Grande (Angra do Reis, Rio de Janeiro, Brazil), and *A.alexi* sp. nov. from the Pedra Branca massif (Rio de Janeiro, Rio de Janeiro, Brazil). Moreover, we describe a second species of *Memoan*—also from the Pedra Branca massif—and amend the diagnosis of this genus to accommodate the new species. We also redescribe *Magnoculusobscurus* and compare it to the remaining amydetine genera to better develop diagnoses of the three genera. We add the two new species of *Amydetes* to the identification key proposed by Silveira and Mermudes (2014a) and propose an identification key to genera of Amydetinae. Finally, we illustrate diagnostic traits for all species included here and provide a distribution map for the three new species.

## ﻿Materials and methods

### ﻿Study areas and sampling

The Serra do Mar mountain range, about 1000 km long, constitutes the most outstanding orographic feature of the Atlantic Forest on the South American continent (Almeida and Carneiro 1998). The southward slopes are constantly subjected to orographic rains, making them an important refuge of biodiversity over evolutionary time scales (Sandel et al. 2011). The material studied here was collected in two conservation units within the Serra do Mar: Pedra Branca State Park, in the Pedra Branca massif and Ilha Grande State Park, on the continental island of Ilha Grande.

The Pedra Branca massif is the largest mountain in the city of Rio de Janeiro. It has a humid, tropical climate without a dry season, with average rainfall of 500–2000 mm from December to March (summer), and 500–1000 mm from June to August (winter). Ilha Grande has a humid, tropical climate with average annual temperatures of 21 °C, ranging between 19.9 °C and 27 °C. Both conservation units are endangered by anthropogenic effects on the Atlantic Forest (Vaz et al. 2021). This lends importance and urgency to knowing the fireflies in that region so as to better protect them.

All specimens mentioned here were captured with Malaise traps (190 × 110 cm with 92% ethanol), by active searches in the field, or borrowed from Brazilian natural history museums.

### ﻿List of collections cited

**DZRJ**Coleção Entomológica Prof. José Alfredo Pinheiro Dutra, Departamento de Zoologia, Universidade Federal do Rio de Janeiro;

**DZUP**Coleção Entomológica Pe. Jesus Santiago Moure, Universidade Federal do Paraná;

**INPA**Instituto Nacional de Pesquisas Amazônicas;

**MNRJ**Museu Nacional, Universidade Federal do Rio de Janeiro;

**MNHN**Muséum National d’Histoire Naturelle;

**MZSP**Museu de Zoologia, Universidade de São Paulo;

**ZMHB** Museum für Naturkunde der Humboldt-Universität.

The species distribution maps were made using QGIS v. 2.18.10 (QGIS.org 2017), and the final figures were assembled and edited in Adobe Photoshop CS6.

### ﻿Morphology and taxonomy

The material studied consists of 228 specimens from MNRJ and DZRJ. We follow the classification system of Martin et al. (2019), and terminology follows Silveira and Mermudes (2014a), Silveira et al. (2016b) and Campello-Gonçalves et al. (2019), except for the hind wing morphology, which follows Lawrence et al. (2021). Three specimens of *A.alexi* sp. nov., *A.marolae* sp. nov., *Ma.obscurus*, and *Me.conani* sp. nov. were entirely dissected, cleared in 10% KOH (24–36 h), then each structure was analyzed and measured under a Leica M205C stereomicroscope. All measurements were made at the point with the greatest width or greatest length. All holotypes and paratypes of the new species described in this article are identified with greaseproof, handwritten labels marked “HOLOTYPE” or “PARATYPE”.

Photographs were taken using Leica Application Suite CV3 automatic image editing program, and figures edited and assembled using Adobe Photoshop CS6 software.

**Figure 1.**
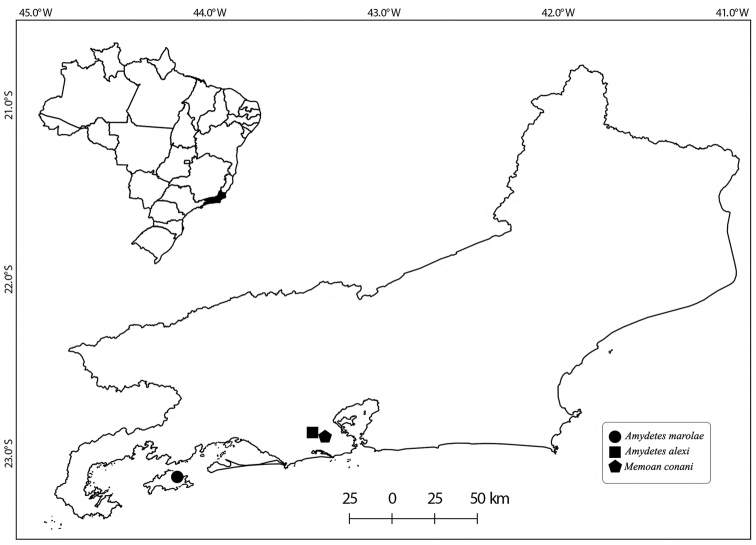
Distribution of new species of Amydetinae in the state of Rio de Janeiro, Brazil, in the southeastern Atlantic Forest.

## ﻿Results

### ﻿Taxonomy

**Family Lampyridae**

#### Subfamily Amydetinae

##### ﻿ Memoan

Taxon classificationAnimaliaColeopteraLampyridae

Silveira & Mermudes, 2013

77759220-95F4-5BE3-8B30-CA7A98B6521B

**Figure 2.**
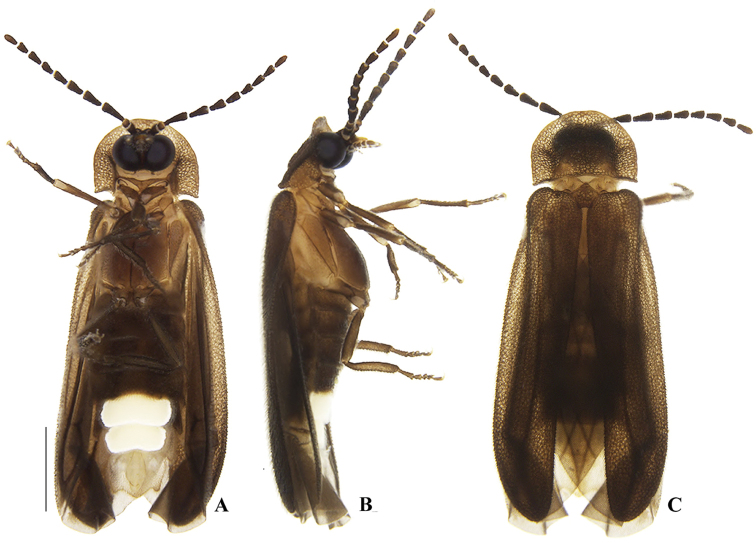
*Memoanconani* sp. nov., male habitus **A** ventral **B** lateral **C** dorsal. Scale bar: 1 mm.

**Figure 3.**
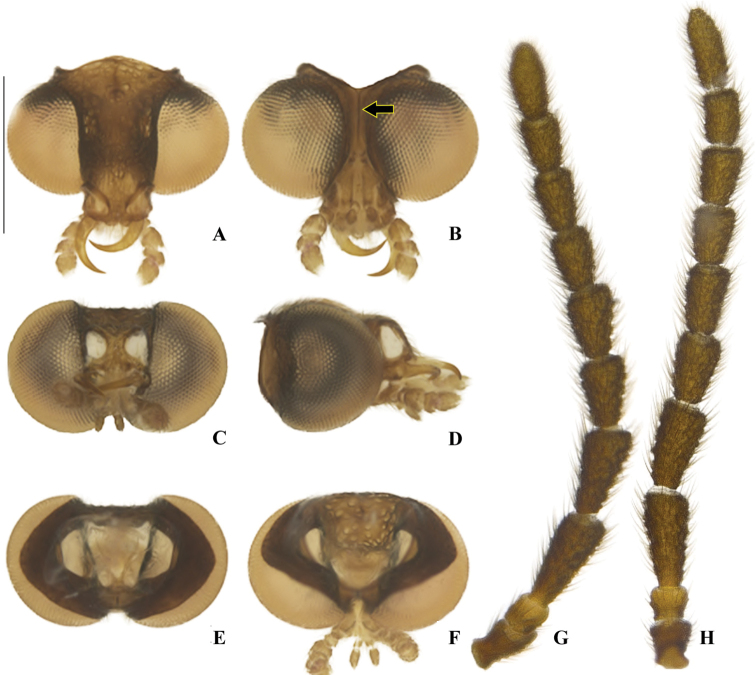
*Memoanconani* sp. nov., head **A** dorsal **B** ventral **C** frontal **D** lateral **E** posterior **F** occipital; antenna **G** dorsal **H** lateral. Scale bar: 0.5 mm (**A–H**). Note the gular margins contiguous (arrow).

**Figure 4.**
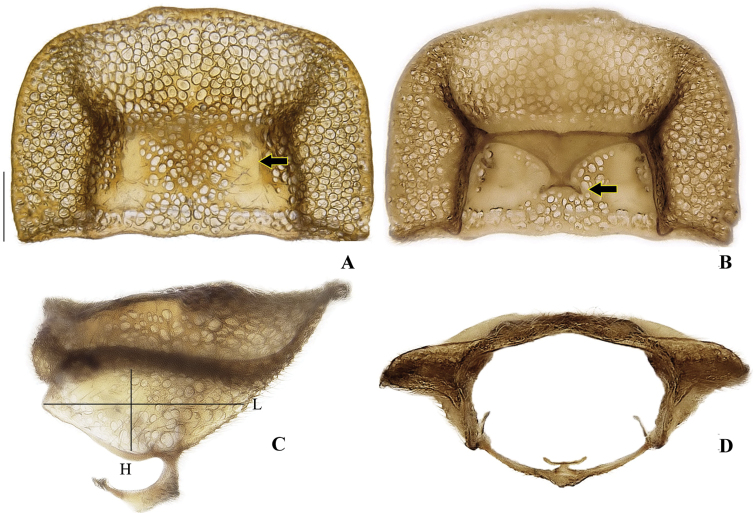
*Memoanconani* sp. nov., pronotum **A** dorsal **B** ventral **C** lateral **D** posterior. Scale bar: 1 mm (**A–D**). Note the L-shaped sclerotized raised tubercles, and proendosternite (arrows). **L**: length; **H**: height.

**Figure 5.**
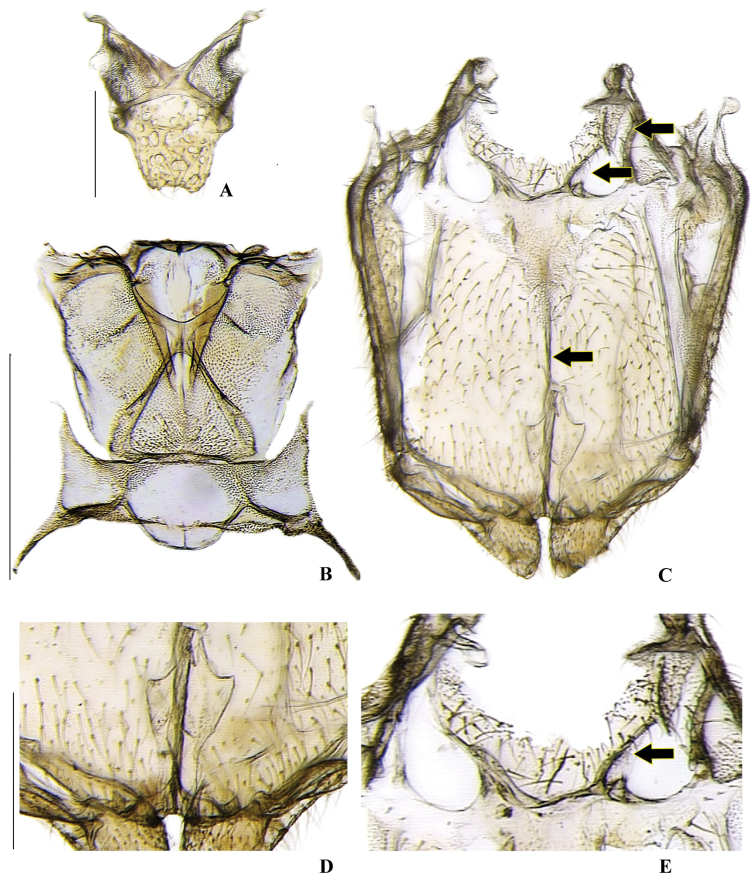
*Memoanconani* sp. nov., mesoscutellum **A** dorsal; metanotum **B** dorsal; pterothorax **C** dorsal; mesoendoesternite **D** dorsal; metaendoesternite **E** dorsal. Scale bars: 200 µm (**A, D–E**); 2 mm (**B, C**). Note the sutures between mesoventrite/mesanespisternum and mesanepisternum/mesepimeron, the metathoracic discrimen, and irregular flap-like projections on mesendosternum (arrows).

**Figure 6.**
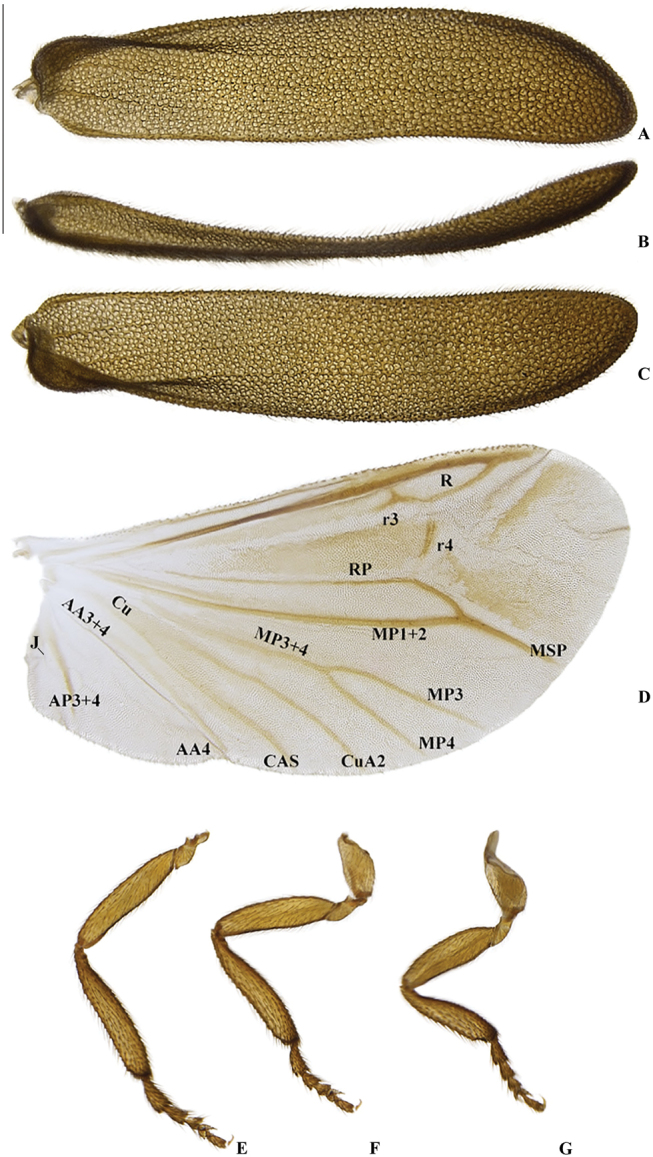
*Memoanconani* sp. nov., elytra **A** dorsal **B** lateral **C** ventral; right wing **D** dorsal; proleg **E** lateral; mesoleg **F** lateral; metaleg **G** lateral. Scale bar: 1 mm (**A–G**).

**Figure 7.**
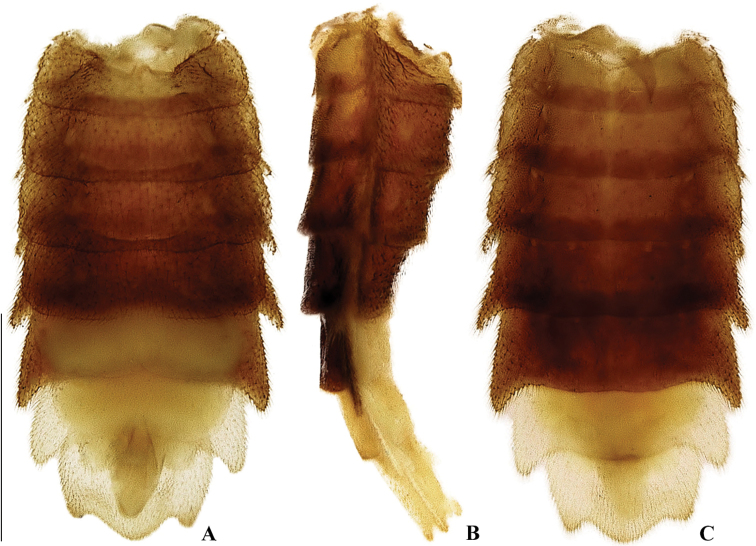
*Memoanconani* sp. nov., abdomen **A** ventral **B** lateral **C** dorsal. Scale bar: 1 mm (**A–C**).

**Figure 8.**
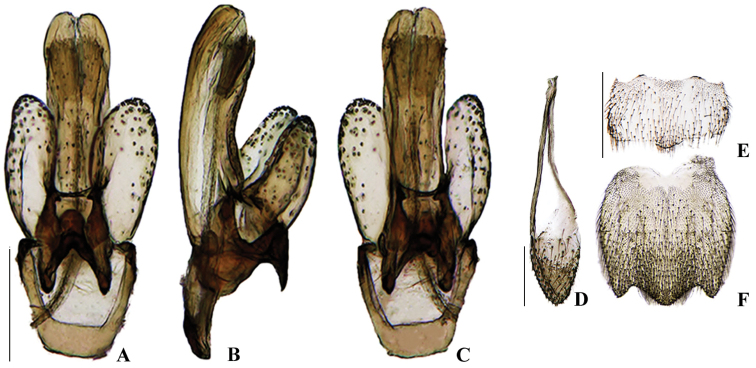
*Memoanconani* sp. nov., aedeagus **A** dorsal **B** lateral **C** ventral; sternum IX **D** ventral; sternite VIII **E** ventral; pygidium **F** dorsal. Scale bars: 200 um (**A–D**); 2 mm (**E, F**).

Figs 2 , 3 , 4 , 5 , 6 , 7 , 8

Memoan Silveira & Mermudes, 2013; Silveira and Mer﻿mudes 2014a: 204; Silveira and Mermudes 2014b: 325; Silveira et al. 2015: 359; Silveira et al. 2016a: 11; Silveira et al. 2016b: 56; Souto et al. 2019: 2; Campello-Gonçalves et al. 2019: 59; Martin et al. 2019: 3; Nunes et al. 2019: 562; Nunes et al. 2020: 4, 7, 8.

###### New diagnosis.

Vertex straight (Fig. 3C); frons convex in lateral view (Fig. 3D); labrum connate to fronto-clypeus, frontoclypeo-labral suture obliterate (Fig. 3C); gular margins contiguous and straight (Fig. 3B); antenna serrate, with ten antennomeres (Fig. 3G, H); pronotum rectangular, with punctures contiguous or separated by 0.1× the puncture width, except for two raised tubercles on the posterior ½ of the disc with sides divergent posteriorly (Figs 4A, 6A); elytron slightly dehiscent (i.e., sinuose inner margin) (Fig. 6A–C); suture between mesanepisternum and mesoventrite visible (Fig. 5C); metathoracic discrimen as long as 3/4 of sternite length (Fig. 5C); lanterns occupying the entire area of sternite VI and VII (Figs 2A, 7A); pygidium with posterior margin bisinuate (Fig. 8E); ventral plate of phallus deeply bilobate (Fig. 8C), phallobase symmetrical (Fig. 8A–C).

###### Redescription.

**Male. *Head*.** Capsule 1.5× wider than long (Fig. 3A); vertex straight (Fig. 3C); eyes separated by 2/3 of head width in frontal view (Fig. 3C) and each eye as wide as 2/3 of head width in dorsal view (Fig. 3A); frons raised and convex in lateral view (Fig. 3D); labrum connate to fronto-clypeus, frontoclypeo-labral suture obliterate (Fig. 3C); gular margins contiguous and straight (Fig. 3B); antennal insertion with outer margin straight, inner margin rounded, as wide as labrum (Fig. 3C). Antenna serrate, with 10 antennomeres (Fig. 3G, H; but may the apical antennomere may be subdivided; see Discussion), as long as 1/2 of body length (Fig. 2A–C); pedicel as long as wide (Fig. 3G); antennomere IV 1.5× longer than III, V–IX subequal in length, X as long as IV (Fig. 3G, H). Maxillary palp with palpomeres I and III 2× wider than long (Fig. 3A–D), II and IV 1.5× wider than long. Labial palp with one or two palpomeres, apical palpomere digitiform (Fig. 3B, F; Silveira and Mermudes 2013: fig. 5). Mentum completely longitudinally divided (Fig. 3B); gular margins contiguous and straight (Fig. 3B). Occipital foramen ellipsoid in posterior view (Fig. 3E, F). ***Thorax*.** Pronotum rectangular (Fig. 4A, B), 2× longer than head length in ventral view (Fig. 2A), 1.5× longer than wide in dorsal view (Fig. 4A), with punctures contiguous or separated by 0.1 puncture width, except for two elongate tubercles on the posterior 1/2 of the disc, sides of tubercles divergent posteriorly (Figs 4A, 6A); hypomeron 2.5× longer than tall (Fig. 4C), with punctures contiguous or separated by 0.1× the puncture width (Fig. 4C); prosternum 0.5× as wide as pronotum in ventral view (Fig. 4B); proendosternite as long as 1/5 of prosternum width (Fig. 4B). Mesocutellum with wide, irregular shaped punctures, contiguous or separated by 0.1× puncture width (Fig. 5A). Elytron slightly dehiscent (i.e. sinuose inner margin), each subparallel-sided (Fig. 6A–C), almost 6× longer than wide. Hind wing (Fig. 6D) with vein r4 poorly sclerotized apically; radial cell poorly sclerotized posteriorly, 2× to 3× wider than long; vein r3 present or absent, vein CuA1 and CuA3+4 present or absent, vein J evanescent as long as 1/4 AP3+4 length (Fig. 6D, Silveira and Mermudes 2013: fig. 8). Metanotum 1.5× wider than long, posterior margin straight, allocrista distinct, well-sclerotized (Fig. 5B). Mesoventrite sclerotized (Fig. 5C), posterior margin rounded; suture between mesanepisternum and mesoventrite visible (Fig. 5C); mesendosternum with irregular flap-like projections (Fig. 5E); metaendosternum spatulate, diamond-shaped (Fig. 5D); metathoracic discrimen as long as 3/4 of sternite length (Fig. 5C). Tibial spurs absent (Fig. 6E–G), procoxa distally constricted, femur as long as tibia, tarsus I>V>II>IV>III (Fig. 6E–G). ***Abdomen*.** Lanterns occupying the entire area of sternite VI and VII (Figs 2A, 7A); sternite VIII 3× wider than long, lateral margins rounded, posterior margin mucronate (Fig. 8E). Sternite IX symmetrical (Fig. 8D), 3× longer than wide, posterior margin rounded. Pygidium as long as wide (Fig. 8F), posterior margin bisinuate, posterolateral angles acute, median 1/3 extending slightly beyond posterolateral angles. Phallus with dorsal and ventral plates overlapping mesal concavity of phallobase (Fig. 8A–C); dorsal plate shorter than ventral plate, ventral plate deeply cleft medially, forming two lobes, apical lobes bent dorsad; parameres symmetrical and broadly rounded at apex in dorsal and lateral views, anterior margin rounded, apically separated from each other and 0.5× shorter than phallus; phallobase symmetrical (Fig. 8A–C).

**Females and immature stages.** Unknown.

###### Remarks.

The discovery of a second species of *Memoan* (see below) called for an updated diagnosis for this genus. *Memoan* is distinguishable from other amydetine genus by their serrate antennae. Silveira and Mermudes (2013) pointed out that *Memoan* shares the following traits with the other amydetine genera: continuous glow (present in *Amydetes* spp. and *Magnoculus* spp.); eyes ventrally close-set (present in some species of *Magnoculus*; Constantin 2011); labial and maxillary apical palpomere with rounded apex (present in *Amydetes* spp. and *Magnoculus* spp.); punctures wide and irregularly outlined on pronotum and scutellum, sometimes on hypomeron as well (widespread in *Magnoculus* spp., absent from disc in *Amydetes* spp.). Recent phylogenetic studies based on molecular data corroborate a placement in Amydetinae (Martin et al. 2019). The evolutionary history of these traits is yet to be assessed.

##### ﻿ Memoan fruhstorferi

Taxon classificationAnimaliaColeopteraLampyridae

comb. nov. (Pic, 1942)

3882C6C6-C9B3-53F8-983C-DF6AD474FC7B

Photinus fruhstorferi Pic, 1942: 16; McDermott 1966: 39
Memoan ciceroi Silveira & Mermudes, 2013: 80 syn. nov.

###### Remarks.

After studying Maurice Pic’s collection at the MNHN, we found that the holotype *P.fruhstorferi* of examined had all the diagnostic traits of *Me.ciceroi* (e.g., antenna with 10 antennomeres, apical antennomere subdivided, labial palp with one palpomere; Silveira and Mermudes 2013). Therefore, we propose that *Me.ciceroi* is a subjective junior synonym of *P.fruhstorferi* Pic, 1942.

Pic (1942: 16) described *P.fruhstorferi* as follows: “*angustatus et elongatus*, *testaceus. Long. 6 m*, *Bresil*, - *Character par sa coloration claire jointe à sa forme élancée*”. The current definition of *Photinus* is controversial, as pointed out by several firefly specialists (e.g., McDermott 1964), and needs taxonomic revision. In fact, *P.fruhstorferi* does not have characters normally found in *Photinus*, such as simple antennae shorter than 1/2 body length, pronotum with sides white and laterally expanded, abdominal tergites rounded, and phallobase relatively long (McDermott 1964). Therefore, we transfer *P.fruhstorferi* to *Memoan*, generating *Me.fruhstorferi* (Pic, 1942) comb. nov. and synonymize it with *Me.ciceroi*, syn. nov., over which it has priority.

###### Material examined.

***Holotype***: Bearing the label: “Espírito Santo. Brasil. ex coll Fruhstorfer.” [aged green label, typewritten]; “TYPE” [aged red label, typewritten]; “R Fruhstorferi” [aged white label, handwritten] (MNHN; Suppl. material 1: Fig. S1).

##### ﻿ Memoan conani

Taxon classificationAnimaliaColeopteraLampyridae

Campello, Vaz, Mermudes & Silveira, 2022 sp. nov.

CDA5ADD0-091F-580F-9C98-BD372C6728BE

http://zoobank.org/E065FDA4-ED8F-493B-B2BD-03E2001860D8

Figs 2 , 3 , 4 , 5 , 6 , 7 , 8

###### Etymology.

The specific epithet *conani* is a masculine noun in the genitive case. The species is named in honor of Mauricio Conan Mendes Correa de Oliveira. Conan was a biology student at the Universidade Federal do Rio de Janeiro, deceased since January 2019, and was a dear friend of the first author.

###### Diagnosis.

Labial palp with two palpomeres (Fig. 3B, F); scape as long as pedicel (Fig. 3G, H); apical antennomere entire, lacking a subdivision or vestigial joint (Fig. 3G, H).

###### Description.

**Male. *Coloration*.** Tegument dark brown (Fig. 2A–C); antennae dark brown (Fig. 3G, H); pronotum and elytra dark brown (Figs 4A, 6A); abdomen with sternites VI–VIII and tergite VIII translucent (Fig. 7A–C); pygidium translucent (Fig. 8F). ***Head*.** Antennal insertions separated by 0.5× socket width in frontal view (Fig. 3C); scape as long as pedicel (Fig. 3G, H); antennomere III 2× longer than pedicel (Fig. 3G, H); apical antennomere entire, lacking a subdivision or vestigial joint (Fig. 3G, H); compare with Silveira and Mermudes 2013: fig. 6). Labial palp with two palpomeres 2-segmented (Fig. 3B, C, F). ***Thorax*.** Hind wing with radial cell 3× wider than long (Fig. 6D). Mesoscutellum with posterior margin almost straight (Fig. 5A). ***Abdomen*.** Phallus 2× longer than parameres (Fig. 8C); parameres with margins evenly rounded and slightly wider than phallus. (Fig. 8 A–C).

**Females and immature stages.** Unknown.

###### Biology.

Twenty-three individuals were collected in hilly areas in the Pedra Branca massif (Fig. 1) at 400 m above sea level. All specimens were collected in a single, shaded humid, approximately 45° slope. Twelve specimens were collected by active search in April 2017 and eleven in April 2019. Males have a continuous green glow and often fly between 0.5 and 3 m above the ground, often in the understory, sometimes reaching up to roughly 7 m above the ground in the forest canopy. Adults are apparently active in the early twilight hours. Males lack the distinctive smell, similar to marzipan frosting, which has been reported for the type species (Silveira and Mermudes 2013). About 2–5 males were observed flying close together in the same visual field.

###### Remarks.

*Memoanconani* sp. nov. is the second species described in the genus. The differences between species are marked: labial palp (with one palpomere in *Me.fruhstorferi* comb. nov. and with two palpomeres in *Me.conani* sp. nov.), which differs morphologically from the all other lampyrids as by Branham (2010); scape (2× longer than pedicel in *Me.fruhstorferi* comb. nov. and as long as pedicel in *Me.conani* sp. nov.); antennae (serrate, with 10 antennomeres in *Me.conani* sp. nov. and *Me.fruhstorferi* comb. nov., but in *Me.fruhstorferi* comb. nov. antennomeres X and XI are connate; see Silveira and Mermudes 2013). Silveira and Mermudes (2013) described the gular suture as biconcave, but, after re-examination of the material, we observed that these are actually straight throughout most of their length, but slightly divergent posteriorly in both species.

Both species of *Memoan* occur in remnants of montane forests, separated from one another by a distance of 400 km: *Me.fruhstorferi* comb. nov. occurs at the Santa Lúcia Biological Station, in Espírito Santo state, whereas *Me.conani* sp. nov. occurs at the Massif of Pedra Branca, Rio de Janeiro state. Since fireflies are poor dispersers (Silveira et al. 2016a), we hypothesize that *Memoan* is endemic to the Atlantic Forest biome, as it has never been found elsewhere in the field despite extensive surveys (Silveira et al. 2020) and is not present in multiple collections in Brazil (DZUP, INPA, MNRJ, MZSP) and abroad (MNHN, ZMHB). This pattern of endemicity is also seen in several other groups of fireflies (e.g., *Amydetes*; *Luciuranus*; *Araucariocladus*; and *Uanauna*).

*Memoanconani* sp. nov. were only seen in a narrow spatial and temporal window; that is, males were only observed and collected in a single slope within the limits of the PEPB at an altitude around 400 m a.s.l., and only in April. Likewise, *Me.fruhstorferi* comb. nov. has only been collected at about 600 m a.s.l., also exclusively in April. Such narrow environmental preferences have been reported for most firefly species occurring in the Atlantic Forest (Silveira et al. 2020). Given the narrow geographic range, both *Me.conani* sp. nov. and *Me.fruhstorferi* comb. nov. may be seriously threatened by habitat loss, like many other Atlantic Forest endemics.

###### Materials examined.

***Holotype***: Brazil • Rio de Janeiro: Rio de Janeiro: Parque Estadual da Pedra Branca, Núcleo Camorim, Trilha do Açude, 22°58'03.7"S, 43°26'45.7"W; 400 m a.s.l.; ♂; 12 Apr. 2017; L. Silveira, L. Campello, S. Vaz, A.L. Diniz leg. (DZRJ). ***Paratypes***: Brazil • same data as for holotype; 10 males (DZRJ) • same data as for holotype; 1 ♂; 9 Apr. 2017; A.L. Diniz leg. (DZRJ) • same data as for holotype; 11 ♂; 13 Apr. 2019; L. Campello, A.L. Diniz, E. Atílio leg. (MNRJ).

#### Genus *Amydetes* Illiger, 1807

##### ﻿ Amydetes marolae

Taxon classificationAnimaliaColeopteraLampyridae

Campello, Vaz, Mermudes & Silveira, 2022 sp. nov.

73CE6E72-96B1-5FC5-A61B-2F1E29907AF0

http://zoobank.org/66B2F83B-E050-43F0-9E1B-2BCC8525DCB5

**Figure 9.**
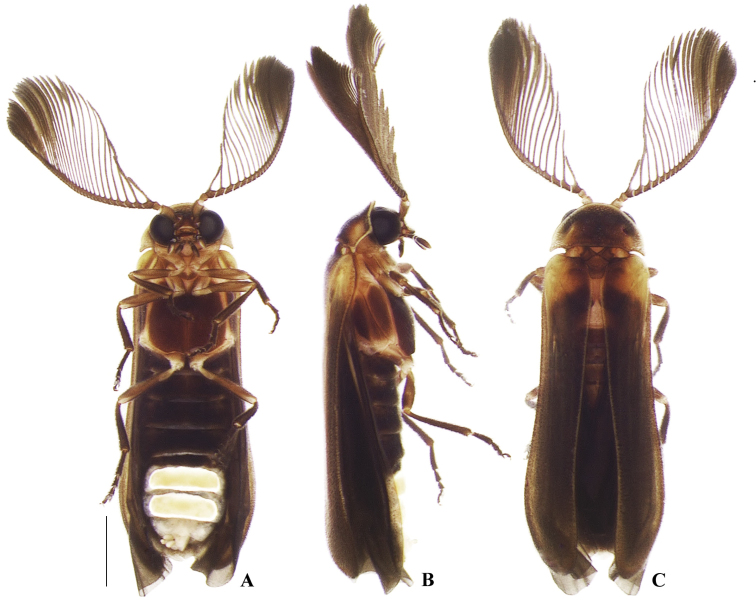
*Amydetesmarolae* sp. nov., male habitus **A** ventral **B** lateral **C** dorsal. Scale bar: 1 mm (**A–C**).

**Figure 10.**
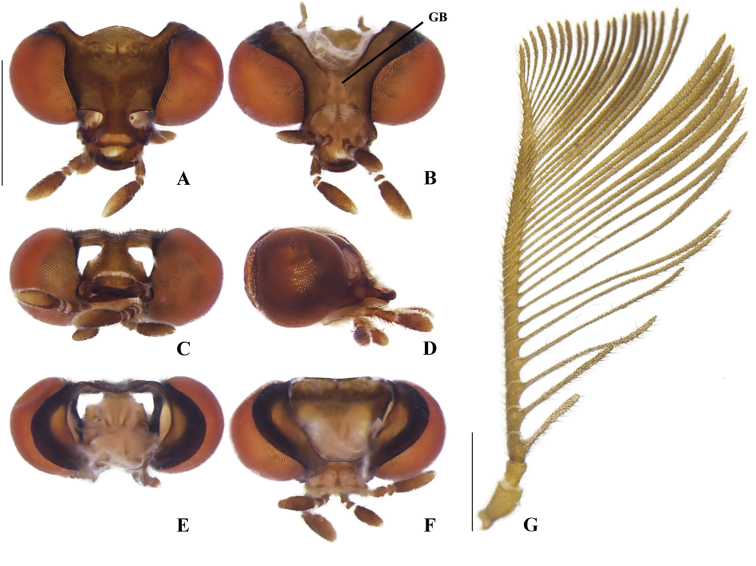
*Amydetesmarolae* sp. nov., head **A** dorsal **B** ventral **C** frontal **D** lateral **E** posterior **F** occipital; antennae **G** dorsal. Scale bars: 1 mm (**A–F**); 2 mm (**G**). **GB**: gular bar.

**Figure 11.**
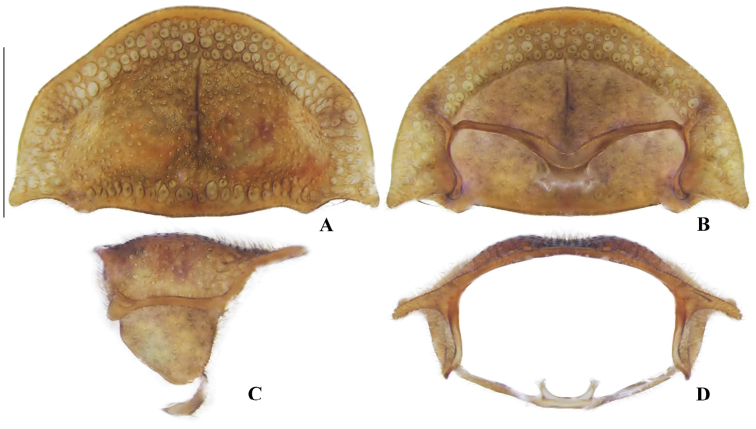
*Amydetesmarolae* sp. nov., pronotum **A** dorsal **B** ventral **C** lateral **D** posterior. Scale bar: 1 mm (**A–D**).

**Figure 12.**
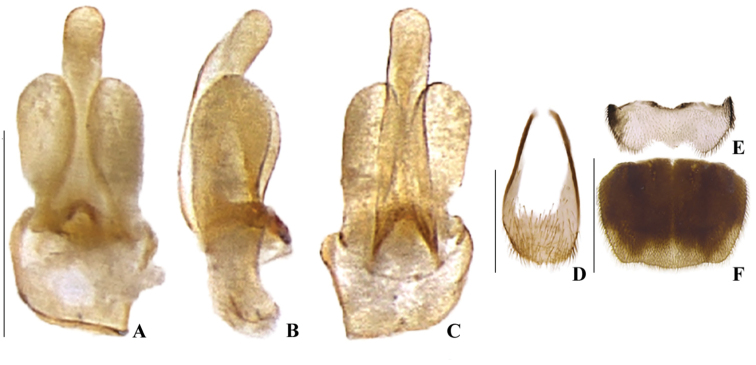
*Amydetesmarolae* sp. nov., aedeagus **A** dorsal **B** lateral **C** ventral; sternum IX **D** ventral; sternite VIII **E** ventral; pygidium **F** dorsal. Scale bars: 0.5 mm (**A–D**); 1 mm (**E, F**).

Figs 9 , 10 , 11 , 12

###### Etymology.

The specific epithet *marolae* is a feminine noun in the genitive case. This species is named in honor of our colleague and friend, Raquel Santos Soares Queiroz, alias “Marola”, who helped us collect the type specimens.

###### Diagnosis.

Antennae with scape and pedicel yellowish brown (Fig. 10G), flagellum light brown (Fig. 10G); pronotum yellowish brown (Fig. 11A–D); abdomen dark brown, with sternites VI–VIII translucent (Figs 9A, 12E), pygidium dark brown with a translucent posterior line (Fig. 12F); antennae with 32–41 antennomeres (Fig. 10G); antennomere III with flabellum 2× longer than antennomere III; antennomere III as long as scape (Fig. 10G); maxillary apical palpomere 10× longer than III (Fig. 10A, B); hypomeron as long as tall (Fig. 11C); sternite VIII with posterior margin bisinuate, central 1/3 slightly shorter than posterolateral angles (Fig. 12E); lanterns occupying almost the entire area of sternite VI and VII (Fig. 9A).

###### Description.

**Male. *Coloration*.** Antennae with scape and pedicel yellowish brown (Fig. 10G), flagellum pale brown (Fig. 10G); pronotum yellowish brown (Fig. 11A–D); abdomen dark brown, with sternites VI– VIII translucent (Figs 9A, 12E), pygidium dark brown with a translucent posterior line (Fig. 12F). ***Head*.** Antennae flabellate with 32–41 antennomeres (Fig. 10G); antennomere III as long as pedicel, with flabellum 2× longer than antennomere III (Fig. 10G). Fronto-clypeus as wide as 1.5× distance between antennifers process (Fig. 10C). Maxillary palpomere I as long as wide; II 2× longer than wide, III approximately 0.1× of IV length (Fig. 10A–F). Gular margins separated by 1/3 maxillary palpomere IV length (Fig. 10B). ***Thorax*.** Pronotum 1.5× wider than head width in ventral view (Fig. 9A), 1.5× wider than long (Fig. 11A, B). Hypomeron as long as tall (Fig. 11C). ***Abdomen*.** Lanterns occupying almost the entire area of sternite VI and VII (Fig. 9A). Sternite VIII with posterior margin bisinuate, central 1/3 slightly shorter than posterolateral angles (Fig. 12E). Pygidium with a translucent posterior line (Fig. 12F). Phallus 1.5× longer than parameres (Fig. 12A–C); parameres basally constricted, as long as phallobase (Fig. 12A); phallobase asymmetrical (Fig. 12A, C).

**Females and immature stages.** Unknown.

###### Biology.

One hundred forty-six individuals were collected in hilly areas of the Ilha Grande State Park (Fig. 1) between 160–660 m a.s.l. in 2017 and 2018. The highest abundance was observed between 160–170 m a.s.l. This species has a continuous blue-green glow. Males fly between 0.1 and 4 m above ground level, bending their abdomens downward, possibly to light the ground in search of females, (as in other *Amydetes* spp.; Vaz et al. 2021); they sometimes flying upwards towards the forest canopy. They are apparently active in the first hours of complete darkness. About 5–10 males were observed flying together in the same visual field.

###### Remarks.

*Amydetesmarolae* sp. nov. is similar to *A.bellorum*, with which it shares the following combination of traits: labrum connected to fronto-clypeus by membrane; maxillary palpomere IV at least 6× longer than III (up to 7× in *A.bellorum*, and at least 10× in *A.marolae* sp. nov.); hypomeron as long as tall; and sternite VIII bisinuate. Nevertheless, *Amydetesmarolae* sp. nov. differs from *A.bellorum* by: body length (average = 0.67 mm [*n* = 10, range = 0.6–0.8] in *A.marolae* and 0.87 mm [*n* = 10, range = 0.8–1.0] in *A.bellorum*); pygidium (entirely dark brown in *A.bellorum*, dark brown with a translucent posterior line in *A.marolae* sp. nov.).

*Amydetesmarolae* sp. nov. occurs in a mountainous and coastal region of the Serra do Mar range, where it is found between 160 and 660 m a.s.l., with greater abundance in parts below to 400 m a.s.l. Despite extensive sampling (e.g., Silveira et al. 2020), *A.marolae* sp. nov. has never been collected anywhere other than Ilha Grande, unlike *A.bellorum* which does not occur on Ilha Grande. Ilha Grande is separated by approximately 3 km from the mainland, which may be an important barrier to dispersal, especially considering that *Amydetes* females are perhaps flightless (Silveira and Mermudes 2014a). As such, *A.marolae* sp. nov. is regarded here as probably endemic to Ilha Grande.

###### Materials examined.

***Holotype***: Brazil • Rio de Janeiro: Angra dos Reis: Parque Estadual da Ilha Grande, Pico do Papagaio; 23°09'05.8"S, 44°11'19.9"W; 660 m a.s.l.; ♂; Apr. 2018; L. Campello, L. Silveira, R. Queiroz, S. Vaz leg. (DZRJ). ***Paratypes***: Brazil • Rio de Janeiro: Angra dos Reis: Parque Estadual da Ilha Grande, Estrada para Dois Rios, Poço do Soldado; 23°10'04.7"S, 44°11'03.5"W; 160 m a.s.l.; 7 ♂; Jul. 2018; L. Campello, L. Silveira, S. Vaz, R. Queiroz leg. (DZRJ) • same data as for preceding; 2 ♂; Sep. 2017 (DZRJ) • same data as for preceding; 2 ♂; Oct. 2017 (DZRJ) • same data as for preceding; 2 ♂; Dec. 2017 (DZRJ) • same data as for preceding; 24 ♂; Mar. 2018 (DZRJ) • same data as for preceding; 11 ♂; Apr. 2018 (DZRJ) • same data as for preceding; 20 ♂; May. 2018 (DZRJ) • same data as for preceding; 11 ♂; Jun. 2018 (DZRJ) • same data as for preceding; 18 ♂; Aug. 2018 (DZRJ) • same data as for preceding; 1 ♂; Nov. 2018 • same data as for preceding; 23°10'05.7"S, 44°11'04.0"W; 170 m a.s.l.; 1 ♂; Sep. 2017; (MNRJ) • same data as for preceding; 1 ♂; Oct. 2017 (MNRJ) • same data as for preceding; 1 ♂; Jan. 2018 (MNRJ) • same data as for preceding; 3 ♂; Apr. 2018 (MNRJ) • same data as for preceding; 7 ♂; May. 2018 (MNRJ) • same data as for preceding; 5 ♂; Jun. 2018 (MNRJ) • same data as for holotype; 23°08'49.9"S, 44°10'51.5"W; 335 m a.s.l.; 1 ♂; Nov. 2017 (MNRJ) • same data as for preceding; 2 ♂; Dec. 2017 (MNRJ) • same data as for preceding; 1 ♂; Feb. 2018 (MNRJ) • same data as for preceding; 1 ♂; Apr. 2018 (MNRJ) • same data as for preceding; 3 ♂; Jul. 2018 (MNRJ) • same data as for preceding; 1 ♂; Aug. 2018 (MNRJ) • same data as for holotype; 23°08'51.5"S, 44°10'52.4"W; 345 m a.s.l.; 3 ♂; Oct. 2017 (MNRJ) • same data as for preceding; 1 ♂; Nov. 2017 (MNRJ) • same data as for preceding; 2 ♂; Jan. 2018 (MNRJ) • same data as for preceding; 1 ♂; Mar. 2018 (MNRJ) • same data as for preceding; 1 ♂; May. 2018 (MNRJ) • same data as for preceding; 5 ♂; Jul. 2018 (MNRJ) • same data as for holotype; 23°08'47.2"S, 44°11'09.4"W; 440 m a.s.l.; 1 ♂; Jun. 2018 (MNRJ) • same data as for preceding; 6 ♂; Aug. 2018 (MNRJ).

##### ﻿ Amydetes alexi

Taxon classificationAnimaliaColeopteraLampyridae

Campello, Vaz, Mermudes & Silveira, 2022 sp. nov.

508EE05A-4924-556F-8645-BEE0291DE41F

http://zoobank.org/D396352E-BD3B-490B-A1EE-AC2A6389B303

**Figure 13.**
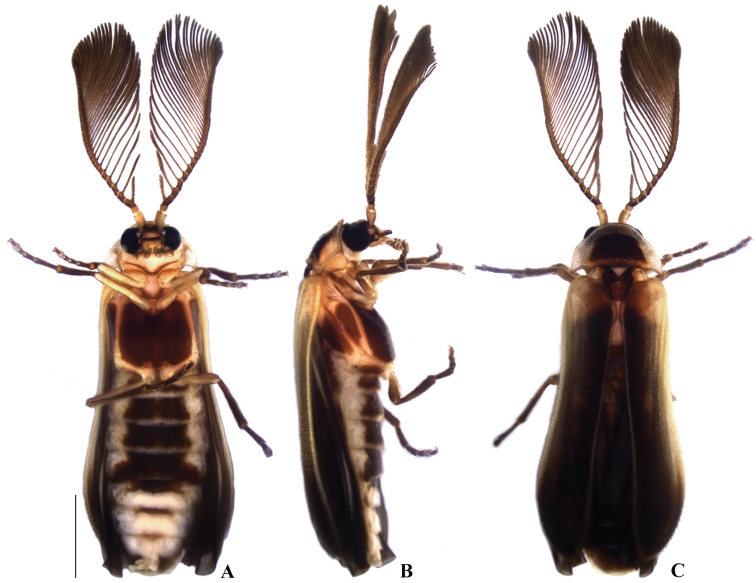
*Amydetesalexi* sp. nov., male habitus **A** ventral **B** lateral **C** dorsal. Scale bar: 2 mm (**A–C**).

**Figure 14.**
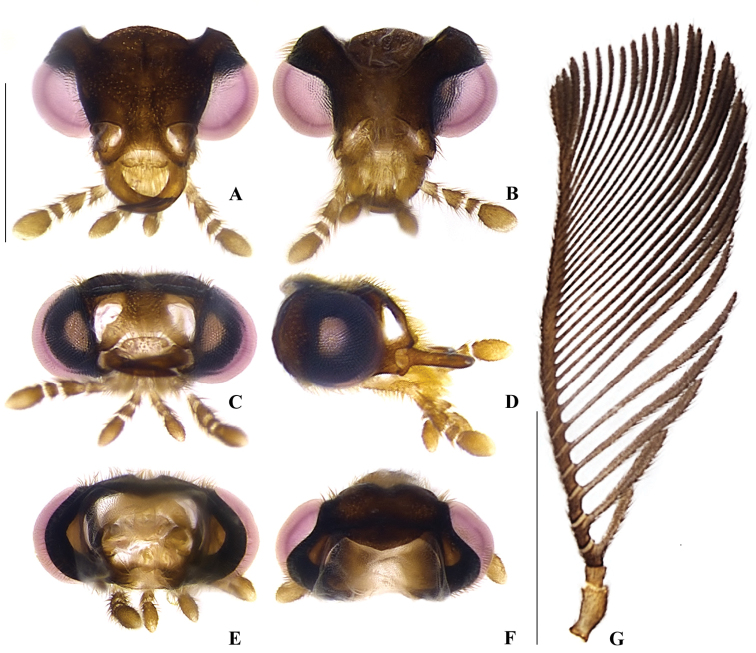
*Amydetesalexi* sp. nov., head **A** dorsal **B** ventral **C** frontal **D** lateral **E** posterior **F** occipital; antennae **G** dorsal. Scale bars: 1 mm (**A–F**); 2 mm (**G**).

**Figure 15.**
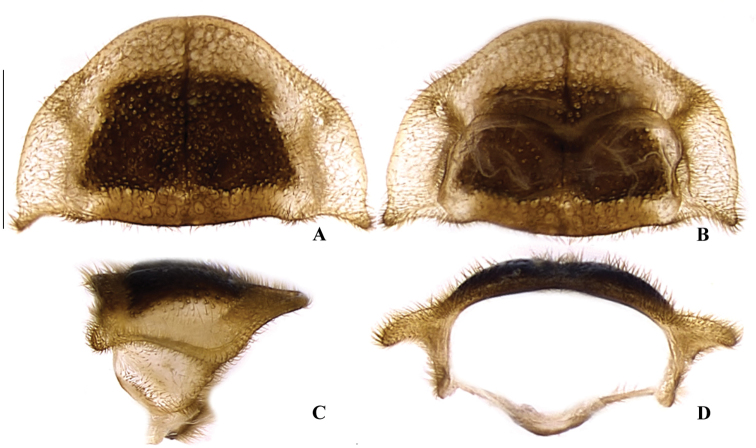
*Amydetesalexi* sp. nov., pronotum **A** dorsal **B** ventral **C** lateral **D** posterior. Scale bar: 1 mm (**A–D**).

**Figure 16.**
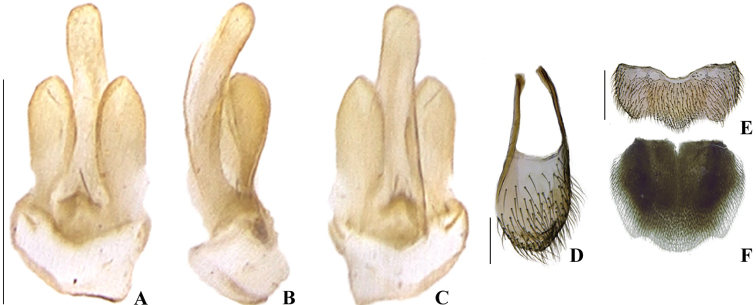
*Amydetesalexi* sp. nov., aedeagus **A** dorsal **B** lateral **C** ventral; sternum IX **D** ventral. sternite VIII **E** ventral; pygidium **F** dorsal. Scale bars: 0.5 mm (**A–D**); 1 mm (**E–F**).

Figs 13 , 14 , 15 , 16

###### Etymology.

The specific epithet *alexi*, is a masculine noun in the genitive case. This species is named in honor of Alex Schomaker Bastos, our dearest friend and biology student at the Universidade Federal do Rio de Janeiro, who was murdered on 8 January 2015.

###### Diagnosis.

Antennae with scape and pedicel yellowish brown (Fig. 14G), flagellum dark brown (Fig. 14G); pronotal disc dark brown with margins yellowish brown (Fig. 15A, B); elytra dark brown with outer margin yellow (Fig. 13A–C); posterolateral margin of thorax yellowish brown (including the posterior corners of metaepisternum and metaepimeron, in addition to the anterior portion of metacoxa; Fig. 13A); legs yellowish brown darkened to dark brown toward apex (Fig. 13A); sides of sternites II–V translucent (Fig. 13A); sternite VIII translucent (Fig. 16E); antennae with 33–37 antennomeres (Fig. 13G); antennomere III 0.5× longer than scape (Fig. 14G); maxillary and labial apical palpomere subequal in length (Fig. 14A–E); hypomeron as long as tall (Fig. 15C); lantern occupying the posterior 1/2 of the sternites VI and VII, and 3/4 the width of these sternites (Fig. 13A).

###### Description.

**Male. *Coloration*.** Antennae with scape and pedicel yellowish-brown (Fig. 14G), flagellum dark brown (Fig. 14G); pronotal disc dark brown with margins yellowish brown (Fig. 15A, B); elytra dark brown with outer margin yellow (Fig. 13A–C); posterolateral margin of thorax yellowish brown (Fig. 13A); legs yellowish brown darkened to dark brown toward apex (Fig. 13A); sides of sternites II–V translucent (Fig. 13A); sternite VIII translucent (Fig. 16E). ***Head*.** Antennae flabellate with 33–37 antennomeres (Fig. 14G); antennomere III 1/2× longer than scape, with flabellum 3× longer than antennomere III. Fronto-clypeus as wide as 1/2 distance between antennifers process (Fig. 14C). Maxillary and labial apical palpomere subequal in length (Fig. 14A–E); maxillary palpomere I, II and IV 2× longer than wide, III 2× wider than long, I 2/3× longer than II, II approximately 2× longer than III, IV approximately 3× longer than III (Fig. 14A–D). Gular margins separated by 1/2 length of maxillary palpomere IV (Fig. 16B). ***Thorax*.** Pronotum 1.5× wider than head width in ventral view (Fig. 13A), 1.5× wider than long (Fig. 15A). Hypomeron as long as tall (Fig. 15C). ***Abdomen*.** Lanterns occupying the posterior 1/2 of sterna VI and VII, as wide as 2/3 the width of these sternites (Fig. 13A). Sternite VIII with posterior margin bisinuate, central 1/3 longer than posterolateral angles (Fig. 16E). Pygidium 1.5× wider than long (Fig. 16F), with posterior margin bisinuate, postero-lateral projections as long as 1/5 length of the central projection. Phallus 1.5× longer than parameres (Fig. 16A–C); parameres basally constricted, 2× longer than phallobase (Fig. 16A–C); phallobase asymmetrical (Fig. 16A, C).

**Females and immature stages.** Unknown.

###### Biology.

Forty-five individuals were collected in hilly areas in the Pedra Branca State Park (Fig. 1), between 140 and 800 m a.s.l. Twelve were collected using Malaise traps: four at 250 m a.s.l. and nine at 280 m a.s.l. All individuals were collected between May and August 2017, which are relatively cooler months. Despite limited sampling, we noticed that the greatest abundance was found around 250–500 m a.s.l., with 23 individuals collected. One specimen, collected on 4 October 2017, was actively flying during daylight (~13:00), suggesting that this species is diurnal, which is congruent with the species’ morphology (i.e., smaller eyes and lanterns; Silveira and Mermudes 2014a).

###### Remarks.

*Amydetesalexi* sp. nov. differs from *A.bellorum* and *A.marolae* sp. nov. in the length of maxillary palpomere IV, which is at least 6× longer than III in *A.marolae* sp. nov. and *A.bellorum*, but only 3× longer than III in three species: *A.itatiaia*, *A.apicalis*, and *A.alexi* sp. nov. *Amydetesalexi* sp. nov. differs from *A.itatiaia* in the length of flabellum of antennomere III (3× longer than pedicel in *A.alexi* sp. nov., but equal in *A.itatiaia*). The new species shares the following traits with *A.apicalis*: pronotum with posterolateral angles pointed but weakly developed; flabellum of antennomere III 3× longer than pedicel; and phallobase asymmetrical. Nevertheless, *A.alexi* sp. nov. differs from *A.apicalis* in having antennae with 33–37 antennomeres (37–44 in *A.apicalis*) and lantern of sternite VI up to 1/2 sternite length (3/5 in *A.apicalis*). The distribution of *A.alexi* sp. nov. is apparently restricted to Pedra Branca massif, as it has not been found nearby by our team (e.g., Silveira et al. 2020).

###### Type material.

***Holotype***: Brazil • Rio de Janeiro: Rio de Janeiro: Taquara, Núcleo Pau da Fome, Rio da Fazenda; 285 m a.s.l.; ♂; 6 Aug.–3 Sep. 2017; A. Diniz leg. (DZRJ).

***Paratypes***: Brazil • Rio de Janeiro: Rio de Janeiro: Taquara, Núcleo Pau da Fome, Trilha para o açude; 1 ♂; 7–10 May. 2017; L. Silveira, A. Diniz leg. (MNRJ) • same data as for preceding, Afluente do Rio Grande, perto da Cachoeira Sete Quedas; 255 m a.s.l.; 4 ♂, 22–19 Jun. 2017; A. Diniz leg. (MNRJ) • same data as for preceding; 3 ♂; 19 Jun.–19 Jul. 2017; A. Diniz leg. (DZRJ) • same data as for preceding, Próximo às ruínas; 5 ♂; 23 Jun.–4 Aug. 2017, A. Diniz leg. (MNRJ) • same data as for preceding, Travessia para o Rio da Prata; 800 m a.s.l.; 3 ♂; 20 Jul. 2017; A. Diniz leg. (MNRJ) • same data as for preceding; 450 m a.s.l.; 8 ♂ 8 Aug.–4 Sep. 2017; L. Silveira, A. Diniz leg. (DZRJ) • same data as for preceding; 300–400 m a.s.l.; 4 ♂; 4 Sep. 2017, L. Silveira leg. (DZRJ) • 22°55'59,7"S, 43°26'29,0"W; 140 m a.s.l., 12 ♂; J. Nessimian, L. Dumas, T. Almeida, B. Genário, L. Diniz leg. (DZRJ) • same data as for holotype; 4 ♂ (MNRJ).

### ﻿Key to adult males of *Amydetes* species based on Silveira and Mermudes (2014a), with new species included

**Table:** 

1	Labrum connate to fronto-clypeus, bisinuate, lobes acute; antennal insertions projected (Silveira and Mermudes 2014a: fig. 221)	***Amydetesbolivari***
–	Labrum free, shape variable, lobes never acute; antennal insertions not projected	**2**
2	Elytra with marginal costa with anterior margin rudimentary and not projected ventrally (Silveira and Mermudes 2014a: fig. 99)	**3**
–	Elytra with marginal costa with anterior margin developed and projected ventrally (Silveira and Mermudes 2014a: fig. 100)	**14**
3	Antennae at least with 55 antennomeres, maxillary palpomere IV less than 1.5× longer than III	***Amydetessolaris***
–	Antennae with 45 antennomeres or less, maxillary palpomere IV at least 2× longer than III	**4**
4	Abdominal sternite VIII with posterior margin medially projected	**5**
–	Abdominal sternite VIII with posterior margin straight or irregular	**9**
5	Maxillary palpomere IV at least 6× longer than III	**8**
–	Maxillary palpomere IV 3× longer than III	**6**
6	Lamella I 3× longer than pedicel; pygidium with median lobe rounded	**7**
–	Lamella I as long as pedicel; pygidium with median lobe beveled	***Amydetesitatiaia***
7	Antennomere III 1/5 longer than pedicel (Silveira and Mermudes 2014a); pygidium with median lobe slightly acuminate, at least 3× longer than lateral lobe	***Amydetesapicalis***
–	Antennomere III 0.5× longer than pedicel (Fig. 14G); pygidium with median lobe slightly rounded, almost as long as lateral lobe	***Amydetesalexi* sp. nov.**
8	Pygidium dark brown with posterior 1/3 white, with posterior margin almost straight; phallobase asymmetrical (Fig. 12A)	***Amydetesmarolae* sp. nov.**
–	Pygidium entirely dark brown, with posterior margin bisinuate (i.e., central 1/3 distinctly projected); phallobase symmetrical	***Amydetesbellorum***
9	Pygidium with median lobe at least bisinuate	**10**
–	Pygidium with median lobe acuminate or rounded (not sinuate)	**13**
10	Labrum bisinuate; abdominal sternite VI with lantern almost as long as this sternite	***Amydetesvivianii***
–	Labrum straight or slightly concave; abdominal sternite VI with lantern with up to 3/5 length of this sternite	**11**
11	Fronto-clypeus with anterior margin almost up to antennal insertions, usually with a discreet median bevel; antennomere III as long as pedicel; pronotum with posterior angle strongly deflexed	***Amydetesvagalume***
–	Fronto-clypeus with anterior margin separated from antennal insertions at least 1/2 antennal socket length; antennomere III longer than pedicel; pronotum with posterior angle straight	**12**
12	Maxillary palpomere IV 1/3 longer than labial palpomere III; abdominal sternite VI with lantern 0.5× as wide as sternite; abdominal sternite IX 1/3 longer than syntergite, parameres separated; phallus without subapical indentations	***Amydetescaetite***
–	Maxillary palpomere IV as long as labial palpomere III; abdominal sternite VI with lantern with less than 1/3 as wide as sternite; abdominal sternite IX 2× longer than syntergite, paramerers touching on the inner margin; phallus with subapical indentations	***Amydeteslucernula***
13	Antennomere III as long as pedicel, pronotum with posterior angle deflexed	***Amydetesmarajoara***
–	Antennomere III at least 1/5 longer than pedicel, pronotum with posterior angle fairly straight	**15**
14	Fronto-clypeus with carinae convergent posterad; pronotum with posterior angle weakly projected; hypomeron 2× longer than tall in lateral view; pygidium with median lobe longer than lateral lobe	***Amydetesagnita***
–	Fronto-clypeus without carinae; pronotum with posterior angle well projected; hypomeron 1.5× longer than tall in lateral view; pygidium with median lobe as long as lateral lobe	***Amydetesgoiana***
15	Antennomere III with flabellum as long as antennomere III	**16**
–	Antennomere III with flabellum notably longer antennomere III	**17**
16	Pronotum with posterior angle deflexed; maxillary palpomere VI 4× longer than III; indentation between lobes median and lateral rudimentary	***Amydetesdiscissa***
–	Pronotum with posterior angle straight; maxillary palpomere VI 2× longer than III; pygidium with lateral lobe weakly developed, acute, indentation between lobes median and lateral weakly developed	***Amydetesmanezinha***
17	Abdominal sternite V with posterior margin concave (Silveira and Mermudes 2014a)	**18**
–	Abdominal sternite V with posterior margin straight (Silveira and Mermudes 2014a)	**19**
18	Gular sutures separated by 1/2 maxillary palpomere IV width; hypomeron 2× longer than tall in lateral view; abdominal sternites with posterior margin strongly concave; abdominal sternite VI with lantern as long as 3/5 sternite length and as wide as 3/5 sternite width	***Amydetesfastigiata***
–	Gular sutures separated by maxillary palpomere IV width; hypomeron 1.5× longer than tall in lateral view; abdominal sternites with posterior margin concave; abdominal sternite VI with lantern almost as long as wide as sternite	***Amydetesfucata***
19	Pronotum with posterior angle obtuse	***Amydeteslucioloides***
–	Pronotum with posterior angle acute	**20**
20	Maxillary palpomere IV 2× longer than III	***Amydetesplaumanni***
–	Maxillary palpomere IV 4× longer than III	***Amydetesluzecu***

#### Genus *Magnoculus* McDermott, 1964

(nec *Megalophthalmus* Leach, 1830 [Crustacea])

##### ﻿ Magnoculus obscurus

Taxon classificationAnimaliaColeopteraLampyridae

Olivier, 1885

D735627D-8820-59FF-B898-9F6FA350434D

**Figure 17.**
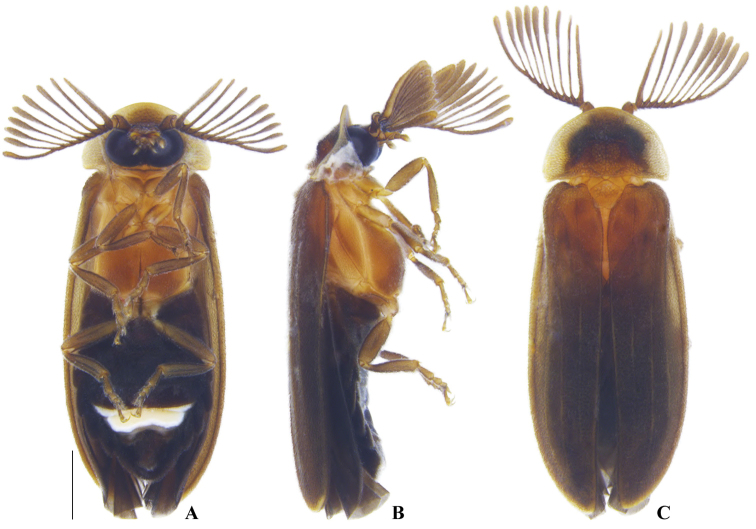
*Magnoculusobscurus*, male habitus **A** ventral **B** lateral **C** dorsal. Scale bar: 1 mm (**A–C**).

**Figure 18.**
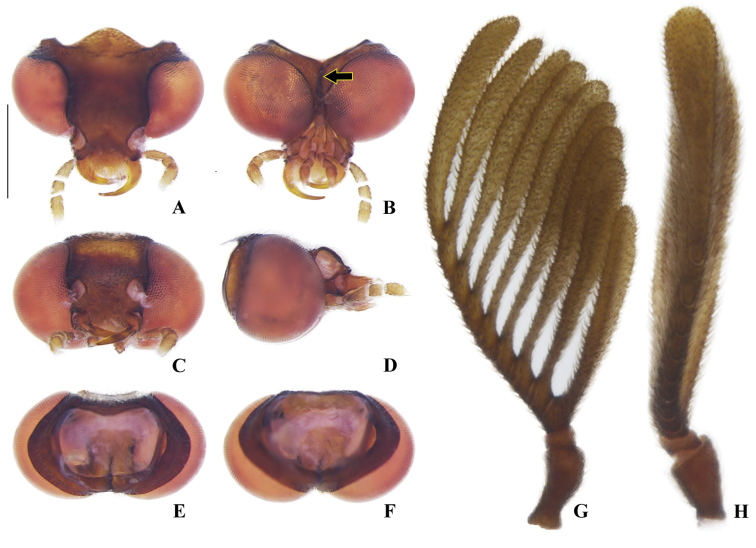
*Magnoculusobscurus*, head **A** dorsal **B** ventral **C** frontal **D** lateral **E** posterior **F** occipital; antennae **G** dorsal **H** lateral. Scale bar: 0.5 mm (**A–H**). Note the gular margins contiguous (arrow).

**Figure 19.**
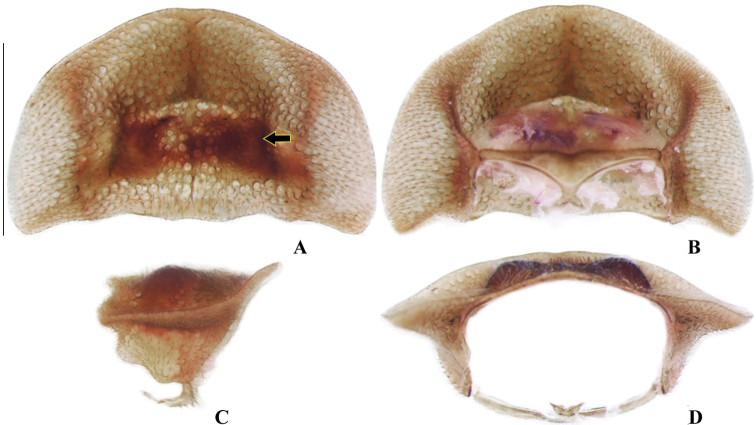
*Magnoculusobscurus*, pronotum **A** dorsal **B** ventral **C** lateral **D** posterior. Scale bar: 1 mm (**A–D**). Note the raised tubercle (arrow).

**Figure 20.**
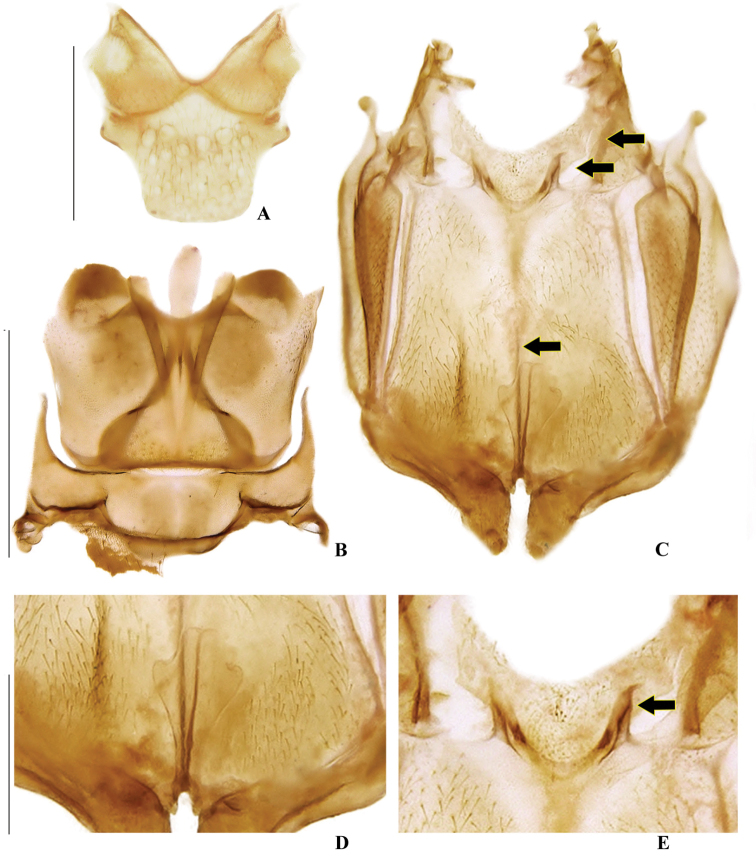
*Magnoculusobscurus*, mesoscutellum **A** dorsal; metanotum **B** dorsal; pterothorax **C** dorsal; mesoendoesternite **D** dorsal; metaendoesternite **E** dorsal. Scale bars: 500 µm (**A, D–E**); 1 mm (**B, C**). Note the sutures between mesoventrite/mesanespisternum and mesanepisternum/mesepimeron; the metathoracic discrimen; and flap-like projections on mesendosternum (arrows).

**Figure 21.**
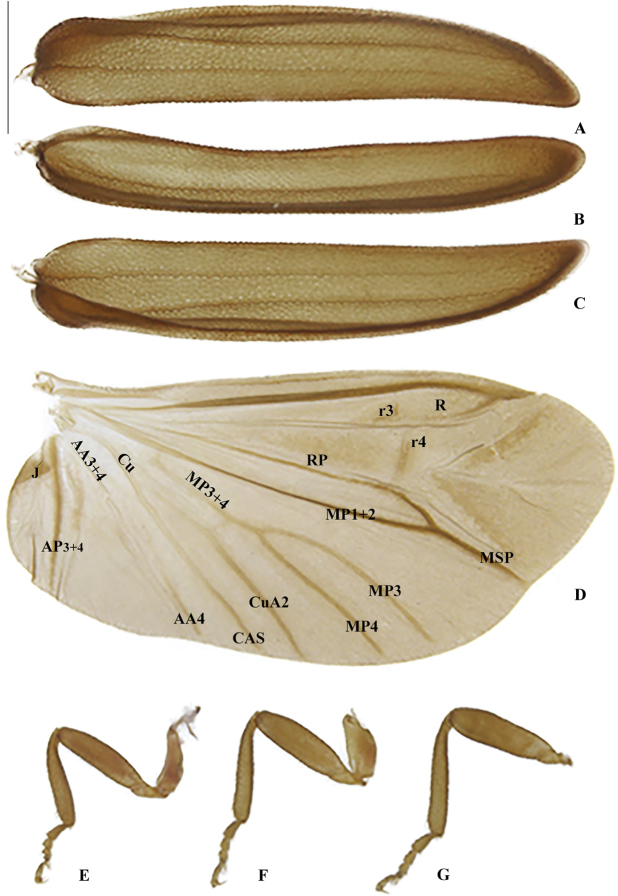
*Magnoculusobscurus*, elytra **A** dorsal **B** lateral **C** ventral; right wing **D** dorsal; proleg **E** lateral; mesoleg **F** lateral; metaleg **G** lateral. Scale bar: 1 mm (**A–G**).

**Figure 22.**
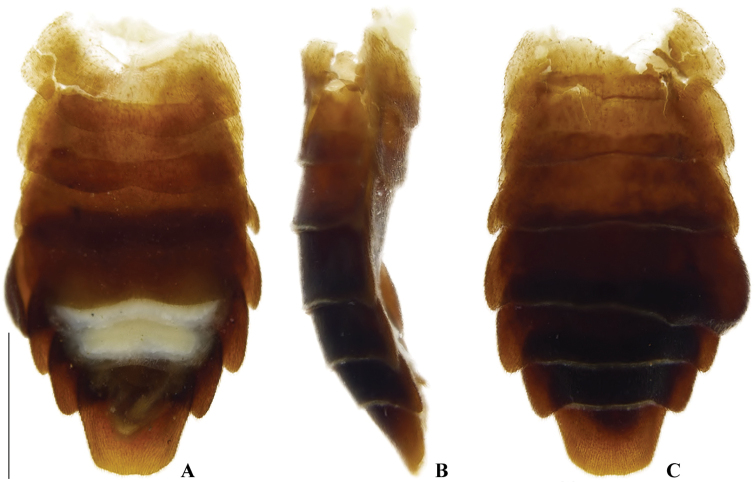
*Magnoculusobscurus*, abdomen **A** ventral **B** lateral **C** dorsal. Scale bar: 1 mm (**A–C**).

**Figure 23.**
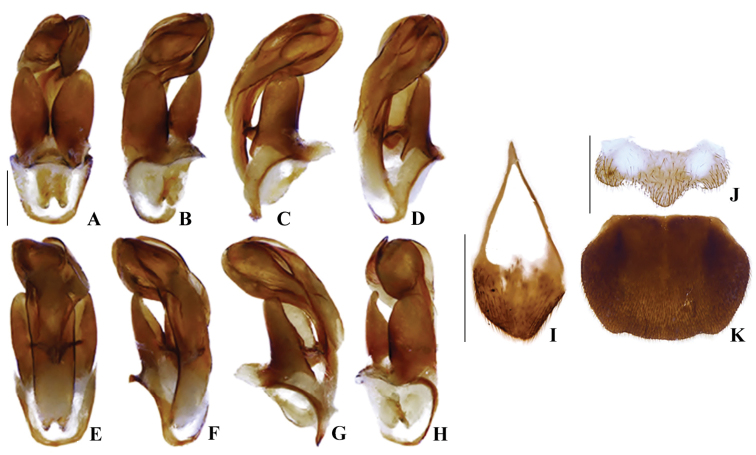
*Magnoculusobscurus*, aedeagus **A** dorsal **B** left dorsolateral **C** lateral **D** left lateroventral **E** ventral **F** right lateroventral **G** lateral **H** right dorsolateral; sternum IX **I** ventral; sternite VIII **J** ventral; pygidium **K** dorsal. Scale bars: 200 µm (**A–H**); 500 μm (**I**); 1 mm (**J, K**).

Figs 17 , 18 , 19 , 20 , 21 , 22 , 23

Megalophthalmus obscurus Olivier, 1885: 146.
Magnoculus obscurus (Olivier, 1885)—Constantin 2011: 153; Silveira et al. 2021: 283, 286, 289.

###### Diagnosis.

Elytra with inner margin dehiscent (i.e., sinuose inner margin) at posterior 1/3 (Fig. 17C); pronotum semilunar, with rounded and obtuse posterolateral angles (Fig. 19A, B), 2× wider than long; frons raised (Fig. 18D); eyes separated by the same length of maxillary palpomere III (Fig. 18B); antennomere III as long as wide (Fig. 18G); antennae shorter than 0.5× body length (Fig. 17A–C); posterior margin of mesoscutellum straight (Fig. 20A); phallobase symmetrical (Fig. 23A–H); sternite IX symmetrical (Fig. 23I); pygidium with posterior margin bisinuate (Fig. 23K).

###### Redescription.

**Male. *Coloration*.** Antennae dark brown (Fig. 18G, H); pronotum brown, with two sclerotized raised tubercles dark brown on the posterior 1/2 (Fig. 19A, B); elytron dark brown (Fig. 21A–C); abdomen completely dark brown (Fig. 22A–C). ***Head*.** Capsule 1.5× wider than long (Fig. 18A); vertex convex (Fig. 18C); eyes separated by 2/3 of head width in frontal view (Fig. 18C); frons raised (Fig. 18D); labrum connate to fronto-clypeus, frontoclypeo-labral suture obliterate (Fig. 18C); antennal insertions rounded (Fig. 18C, D), separated by 0.5× labrum width. Antennae flabellate, lamellae stiff and laterally compressed, with 11 antennomeres (Fig. 18G, H), as long as 1/3 of body length (Fig. 17A–C); pedicel 2× wider than long (Fig. 18G, H), III–V subequal in length, V–IX subequal in length, IX–X subequal in length. Maxillary palp with four palpomeres (Fig. 18B), II–IV subequal in length, III 2× longer than I. Labial palp with three palpomeres (Fig. 18B), I–II subequal in length, III 2× longer than II. Occipital foramen subcordiform in posterior view (Fig. 18E, F). ***Thorax*.** Pronotum semilunar (Fig. 19A–D), almost 2× longer than head length in ventral view (Fig. 17A), 2× wider than long in dorsal view (Fig. 19A), with contiguous and equidistant punctures along the entire surface, except for two sclerotized raised tubercles on the posterior 1/2, equidistant from sides (Fig. 19A, D); hypomeron 2× longer than tall (Fig. 19C); prosternum wider than 1/5 of pronotum width in dorsal view (Fig. 19B). Elytron slightly dehiscent (i.e., sinuose inner margin), subparallel-sided (Fig. 21A–C). Hind wing (Fig. 21D) with radial cell evanescent, 2× wider than long; vein r3 as long as 1/4 r4 length, vein CuA1 absent; vein CuA3+4 present; vein J as long as 1/3 the length of vein AP3+4. Metanotum as long as wide, posterior margin emarginate medially, allocrista distinct (Fig. 20B). Mesoventrite sclerotized (Fig. 20B), posterior margin rounded; suture between mesoventrite and mesanepisternum visible (Fig. 20C), mesendosternum with two irregular, flap-like projections (Fig. 20E); metaendosternum spatulate, diamond-shaped (Fig. 20D). Tibial spurs absent (Fig. 21E–G), tarsus I >V >IV>II=III (Fig. 21E–G). ***Abdomen*.** Lanterns occupying almost the entire area of sternite VI and VII (Fig. 17A); sternites II–VII with sides rounded (Fig. 22A), II, III, VI and VII with posterior margin medially emarginate; sternite VIII mucronate (Fig. 23J); sternite IX symmetrical (Fig. 23I), 1.5× longer than wide, posterior margin rounded. Pygidium 1.5× wider than long (Fig. 23K), sides rounded, posterior margin bisinuate (Fig. 8F), posterolateral angles obtuse, central 1/3 extending slightly longer than posterolateral angles (Fig. 23K). Phallus with a dorsal and ventral plate (Fig. 23H, I) dorsal plate acuminate from apical 1/2 on, skewed to the anatomical left (i.e. left of the specimen), extending about a 1/4 longer than ventral plate towards the base (Fig. 23A, B, F, G), ventral plate bilobed (Fig. 23B–E), lobes spoon-shaped and separated by a deep cleft reaching basal 2/5, projected dorsolaterally to right under dorsal plate, dorsal plate extending a 1/3 beyond parameres; parameres symmetrical and spatulate (Fig. 23A, D, F, H), anterior margin rounded, apically separated from each other (i.e., not fused), and 1/2 shorter than the phallus (Fig. 23A); phallobase symmetrical (Fig. 23A).

**Females and immature stages.** Unknown.

###### Biology.

Fourteen individuals were collected in hilly areas in Ilha Grande State Park between 160–345 m a.s.l. Six specimens were collected using Malaise traps: three at 160 m a.s.l. in August 2017, December 2017, and January 2018; two at 170 m a.s.l. in November 2017 and April 2018; one at 345 m a.s.l. in August 2017. In addition, nine specimens were collected by active searches in July 2017 (five specimens), September 2017 (two specimens), and July 2018 (two specimens). *Ma.obscurus* has a yellowish-green glow, and males often fly between 0.1 and 4 m a.s.l., sometimes reaching up to approximately 7 m a.s.l. towards the forest canopy. Adults are apparently active in the early twilight hours. About five to ten males were observed flying close together in the same visual field.

###### Remarks.

*Magnoculusobscurus* is the second species of genus to be redescribed (see Zaragoza-Caballero 1995 for redescription of Ma.cf.guatemalae). The few species recently studied and illustrated in detail by Constantin (2011) and Zaragoza-Caballero (1995) allowed us to find interesting commonalities and differences among *Magnoculus* species. For example, *Ma.obscurus* shares the following characteristics with its congeners: elytra with three raised costae; pronotum with two sclerotized raised tubercles on the posterior 1/2 and posterior angles obtuse; and sternite VIII mucronate.

A comparison of *Ma.obscurus* with species described and illustrated in detail by Constantin (2011) and Zaragoza-Caballero (1995) is given here, as follows: antennae smaller than 1/2 body length, similar to *Ma.dalensi* and *Ma.touroulti*, but longer than 1/2 body length in *Ma.brulei*, *Ma.dewynteri*, *Ma.poirieri*, and *Ma.guatemalae*; flabellum of antennomere III as long as pronotum length, similar to *Ma.touroulti* and *Ma.guatemalae*, but 0.5× shorter in *Ma.dalensi*, ~2.5× longer in *Ma.brulei*, *Ma.poirieri*, and *Ma.dewynteri*; eyes ventrally close-set in *Ma.obscurus*, similar to *Ma.touroulti* and *Ma.brulei*, but not in *Ma.guatemalae* and *Ma.dewynteri* (undescribed in *Ma.dalensi* and *Ma.poirieri*); gular margins contiguous in *Ma.obscurus*, *Ma.guatemalae*, and *Ma.touroulti*, but open in *Ma.dewynteri* (no information for *Ma.dalensi*, *Ma.poirieri*, and *Ma.brulei*); pronotum semilunar, 2× wider than long, similar to *Ma.touroulti*, *Ma.brulei*, and *Ma.guatemalae*, but as wide as long in *Ma.dalensi*, triangular and as wide as long with anterior margin rounded in *Ma.poirieri* and *Ma.dewynteri*, completely yellow in *Ma.poirieri*, brown with an orange anterior spot in *Ma.dewynteri*, and completely dark brown in *Ma.obscurus*, *Ma.brulei*, *Ma.dalensi*, and *Ma.tourolti*. Pronotal disc with two sclerotized raised tubercles on the posterior 1/2, similar to *Ma.brulei*, *Ma.poirieri*, *Ma.dewynteri*, and *Ma.tourolti*, which are reduced in *Ma.dalensi*. Pygidium bisinuate in *Ma.obscurus*, as in *Ma.dalensi* and *Ma.guatemalae*, but semilunar in *Ma.brulei*, *Ma.dewynteri*, *Ma.poirieri*, and *Ma.touroulti*. Aedeagus of *Ma.obscurus* asymmetrical, as in *Ma.guatemalae*, *Ma.poirieri*, and *Ma.touroulti*, but symmetrical in *Ma.brulei*, *Ma.dewynteri*, and *Ma.dalensi*.

###### Lectotype (designated herein).

Bearing the labels: “SYNTYPE. Megalophthalmus. obscurus. Olivier, 1885. MNHN, Paris-co E. Olivier” [aged red label, typewritten]; “Muséum Paris. Coll. E. Olivier” [green label, typewritten]; “Brésil.” [aged white label, typewritten]; “Sahlberg.” [aged white label, typewritten]; “obscurus. Brésil ‘oliv’ [aged white label with green margins] (MNHN, ex. coll E. Olivier; Suppl. material 1: Fig. S2).

Olivier mentioned examining specimens in his and also in Oberthür’s collections. It is unclear how many specimens Olivier examined, and we did not have the opportunity to carefully look for them at the MNHN.

###### Other materials examined.

Brazil • Rio de Janeiro: Angra dos Reis: Parque Estadual da Ilha Grande), Pico do Papagaio; 4 ♂; 29 Jul. 2017; L. Silveira leg. (DZRJ) • 23°08'51.5"S, 44°10'52.4"W; 345 m a.s.l.; 1 ♂; Aug. 2017 (DZRJ) • 23°10'05.7"S, 44°11'04.0"W; 170 m a.s.l.; 1 ♂, Sep. 2017 (MNRJ) • same data as for preceding; 1 ♂, Apr. 2018 (MNRJ) • same data as for preceding, Estrada para Dois Rios; 2 ♂; 22 Nov. 2017 (MNRJ) • same data as for preceding; 2 ♂; Jul. 2018 (MNRJ) • same data as for preceding, Poço do Soldado; 23°10'04.7"S, 44°11'03.5"W; 160 m a.s.l.; 1 ♂; Dec. 2017 (DZRJ) • same data as for preceding; 1 ♂; Aug. 2017 (MNRJ) • same data as for preceding; 1 ♂; Jan. 2018 (MNRJ).

### ﻿Key to genera of adult male Amydetinae

**Table:** 

1	Antenna with at least 23 antennomeres (Figs 10G, 14G); pronotal disc with punctures contiguous or separated by 1–5× the puncture width, without sclerotized raised tubercles (Figs 11A, 15A); labrum connected to fronto-clypeus by membrane (Figs 10C, 14C; but connate in *Amydetesbolivari* Silveira & Mermudes, 2014)	***Amydetes***
–	Antenna with 10 or 11 antennomeres (Figs 3G, H; 18G, H); pronotal disc with punctures contiguous or separated by 0.1× puncture width, with sclerotized raised tubercles; labrum connate to fronto-clypeus, frontoclypeo-labral suture obliterate (Figs 3C, 18C)	**2**
2	Antenna flabellate (Fig. 18G, H); labial palp with three palpomeres (Fig. 18A, B); pronotum semilunar (Fig. 19A, B); metathoracic discrimen 0.5× longer than sternite length (Fig. 20C)	***Magnoculus***
–	Antenna serrate (Fig. 3G, H); labial palp with one or two palpomeres (Fig. 3B, F; Silveira and Mermudes 2013: fig. 5); pronotum rectangular (Fig. 4A, B); metathoracic discrimen 3/4 longer than sternite length (Fig. 5C)	***Memoan***

## ﻿Discussion

Since Martin et al. (2019) moved *Memoan* to Amydetinae, a more comprehensive subfamily-level diagnosis has been missing for this genus because descriptions comparable to those for other amydetine genera were lacking for *Magnoculus*. Here, we suggest the following combination of characters: if lanterns are present (on sternite VI–VII), then, with a continuous blue-greenish glow (i.e. it never turns off); punctures wide (distance among punctures smaller than puncture diameter), present at least on the lateral expansions, often on the disc as well (Figs 4A, 11A, 15A, 19A); maxillary and labial apical palpomeres with anterior margin rounded (Figs 3C, 10C, 14C, 18C); frons raised (Figs 3D, 10D, 14D, 18D); gular margins distinct, subparallel, separated by submentum width or fused (Figs 3B, 10B, 14B, 18B); tibial spurs absent (Figs 6E–G, 21E–G); hind wing with radial cell evanescent posteriorly; MP3 with proximal part evanescent, CuA1 and CuA3+4 crossveins present or absent (Figs 6D, 21D); phallus with dorsal and ventral plates, longer than parameres, parameres apically rounded (Figs 8A–C, 12A–C, 16A–C, 23A–H). Nevertheless, a more comprehensive comparison with an increased sampling of *Magnoculus* is needed to improve the understanding of the amydetines. Based on the available literature (i.e., Zaragoza-Caballero 1995; Constantin 2011) and the present study, we suggest the following set of diagnostic characters for *Magnoculus*: antenna flabellate (Fig. 18G, H); pronotum with two sclerotized raised tubercles (Fig. 19A), posterior angles obtuse (Fig. 19A, B); elytra with three raised costae (Fig. 21A); sternite VIII with posterior margin bisinuate.

When comparing the three amydetine genera, a few interesting similarities and differences are evident (summarized in Table 1). For example, *Magnoculus* and *Amydetes* have flabellate antennae, which are serrate in *Memoan*; the antennae have10 antennomeres in *Memoan* and *Magnoculus*, and at least 23 antennomeres in *Amydetes*. The Amydetinae also feature the widest range of antennomere variation in the family with 23–62-antennomeres (Nunes et al. 2020). However, all known *Magnoculus* species have 11 antennomeres, and *Memoan* species have 10 (Fig. 3G, H), although *Me.fruhstorferi* comb. nov. has a vestigial joint on the apical most antennomere (suggestive of an ancestral 11 antennomeres antenna), and a 10 antennomeres antenna is likely a derived state of the *Memoan* lineage, since such a state is not observed in phylogenetically close lineages (Nunes et al. 2020). *Amydetes* is the firefly genus with the widest intraspecific variation in the number of antennomeres. In fact, individuals may feature asymmetrical antennomere counts (i.e., differences between the left and right antennae of the same individual; Nunes et al. 2020). *A.marolae* sp. nov. has 32–41 antennomeres, and a variation between the right and left antennae of up to two antennomeres (*n* = 10). In turn, *A.alexi* sp. nov. presents a variation of 33–37 antennomeres, and also a maximum of two antennomeres between the right and left (*n* = 10).

**Table 1.** Comparative table for Amydetinae genera.

	*Amydetes*	*Memoan*	*Magnoculus*
Antennae	Flabellate, with 23–62 antennomeres; antennal sockets reniform	Serrate, with 10 antennomeres; antennal sockets with inner margin straigth and outer margin rounded	Flabellate, with 11 antennomeres; antennal sockets rounded
Fronto-clypeal suture	Connected by membrane	Connate	Connate
Mouthparts	Labial palp with three palpomeres	Labial palp with one or two palpomeres	Labial palp with three pelpomeres
Gula	Gular margins separated by length of labial palpomere II	Gular margins contiguous	Gular sutures contiguous
Pronotum	semilunar, without two sclerotized raised tubercles on the 1/2 posterior; disc with fine punctures	Rectangular; two sclerotized raised tubercles on the 1/2 posterior; disc with deep punctures	Semilunar; two sclerotized raised tubercles on the 1/2 posterior, with or without deep punctures
Elytron	Outer margin emarginate	Outer margin rounded	Outer margin rounded; with three raised costae
Thorax	Metathoracic discrimen as long as 3/4 of sternum length	Metathoracic discrimen as long as 1/2 of sternum length	Metathoracic discrimen as long as 3/4 of sternum length
Aedeagus	Phalobase symmetrical or asymmetrical	Phalobase symmetric	Phalobase symmetric or asymmetric

The pronotum has two sclerotized raised tubercles in *Magnoculus* and *Memoan*, which are absent in *Amydetes*, and there are wide punctures with little interstices in the three genera, although in *Amydetes* these are restricted to the pronotal expansions. The pronotum is semilunar in *Magnoculus* and *Amydetes*, but rectangular in *Memoan*. The eyes are ventrally close-set in both *Memoan* spp., but only in some species of *Amydetes* and *Magnoculus*. Finally, the phallobase is symmetrical in both *Memoan* spp., but may be symmetrical or asymmetrical in *Amydetes* and *Magnoculus* species.

*Memoanconani* sp. nov., *Amydetesmarolae* sp. nov., and *Amydetesalexi* sp. nov. are three new additions to the subfamily Amydetinae recently collected in protected areas. All three species appear to have narrow spatial and temporal distributions and are thought to be endemic to their localities, as known for other amydetine species (e.g., Silveira and Mermudes 2013, 2014a; Constantin 2011). *Amydetesmarolae* sp. nov. and *A.alexi* sp. nov. are present in sympatry with other species of the genus at their localities, while the two species of the genus *Memoan* are in allopatry.

## Supplementary Material

XML Treatment for Memoan

XML Treatment for Memoan fruhstorferi

XML Treatment for Memoan conani

XML Treatment for Amydetes marolae

XML Treatment for Amydetes alexi

XML Treatment for Magnoculus obscurus

## Supplementary materials

Supplementary material 1 Figures S1, S2 Data type pdf file Explanation note Original labels of the type species of *Magnoculusobscurus* and *Memoanfruhstorferi* comb. nov. deposited in the MNHN. https://binary.pensoft.net/file/720432This dataset is made available under the Open Database License (http://opendatacommons.org/licenses/odbl/1.0/). The Open Database License (ODbL) is a license agreement intended to allow users to freely share, modify, and use this Dataset while maintaining this same freedom for others, provided that the original source and author(s) are credited.Lucas Campello, Stephanie Vaz, José R. M. Mermudes, André L. D. Ferreira, Luiz F. L. Silveira
