# Supplementary material for: ﻿Comparative morphology and key to Amydetinae genera, with description of three new firefly species (Coleoptera, Lampyridae)
Source: Zookeys. 2022 Jul 27;1114:131–66. doi: 10.3897/zookeys.1114.77692 (PMC9848971; doi:10.3897/zookeys.1114.77692)
Supplement: Supplementary material 1 — Figures S1, S2 [file zookeys-1114-131_article-77692__-s001.pdf]

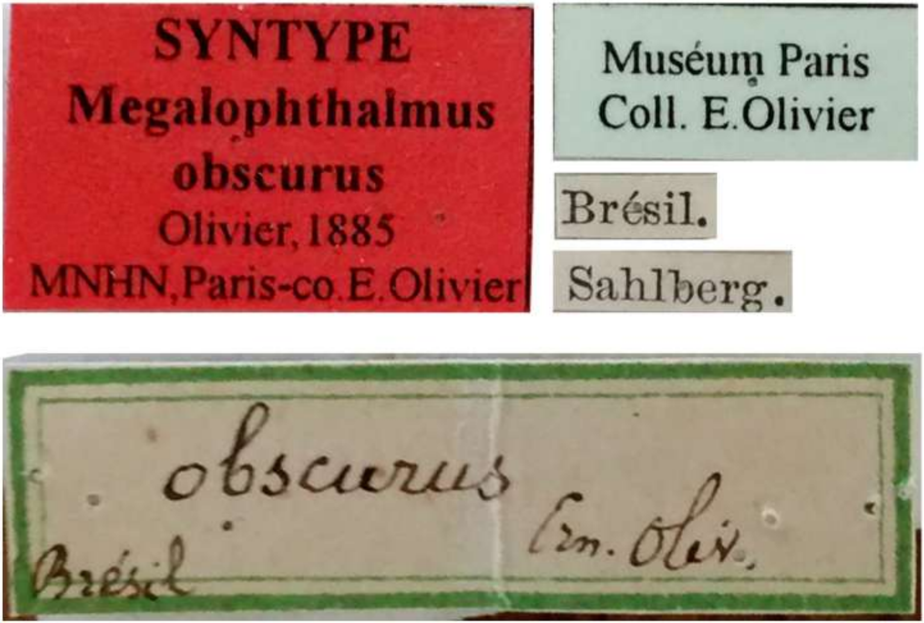
SYNTYPE
Megalophthalmus
obscurus
Olivier, 1885
MNHN, Paris-co. E. Olivier
Muséum Paris
Coll. E. Olivier
Brésil.
Sahlberg.
obscurus
Ern. Oliv.
Brésil

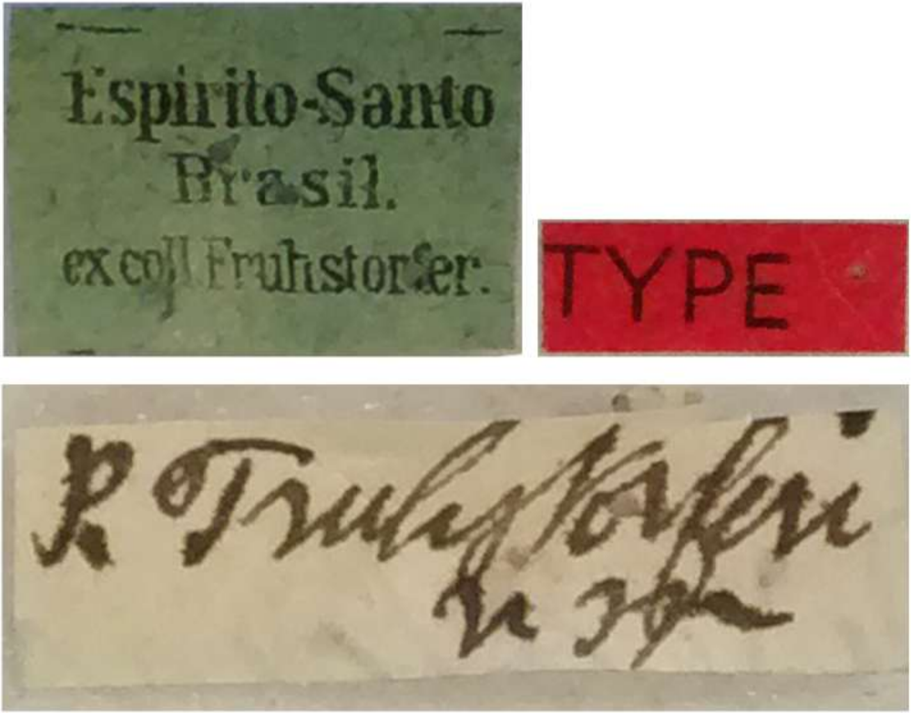
Espirito-Santo
Brasil.
ex coll Fruhstorfer.
TYPE
